# Primate-specific transposable elements shape transcriptional networks during human development

**DOI:** 10.1038/s41467-022-34800-w

**Published:** 2022-11-23

**Authors:** Julien Pontis, Cyril Pulver, Christopher J. Playfoot, Evarist Planet, Delphine Grun, Sandra Offner, Julien Duc, Andrea Manfrin, Matthias P. Lutolf, Didier Trono

**Affiliations:** 1 Laboratory of Virology and Genetics, School of Life Sciences, Ecole Polytechnique Fédérale de Lausanne (EPFL), 1015 Lausanne, Switzerland; 2 Laboratory for Stem Cell Bioengineering, School of Life Sciences, Ecole Polytechnique Fédérale de Lausanne (EPFL), 1015 Lausanne, Switzerland

**Keywords:** Gastrulation, Embryonic stem cells, DNA transposable elements, Genome evolution

## Abstract

The human genome contains more than 4.5 million inserts derived from transposable elements (TEs), the result of recurrent waves of invasion and internal propagation throughout evolution. For new TE copies to be inherited, they must become integrated in the genome of the germline or pre-implantation embryo, which requires that their source TE be expressed at these stages. Accordingly, many TEs harbor DNA binding sites for the pluripotency factors OCT4, NANOG, SOX2, and KLFs and are transiently expressed during embryonic genome activation. Here, we describe how many primate-restricted TEs have additional binding sites for lineage-specific transcription factors driving their expression during human gastrulation and later steps of fetal development. These TE integrants serve as lineage-specific enhancers fostering the transcription, amongst other targets, of KRAB-zinc finger proteins (KZFPs) of comparable evolutionary age, which in turn corral the activity of TE-embedded regulatory sequences in a similarly lineage-restricted fashion. Thus, TEs and their KZFP controllers play broad roles in shaping transcriptional networks during early human development.

## Abstract (web summary)

The human genome harbors more than 4.5 million transposable element (TE)-derived insertions, the result of recurrent waves of invasion and internal propagation. Here they show that TEs belonging to evolutionarily recent subfamilies go on to regulate later stages of human embryonic development, notably conditioning the expression of genes involved in gastrulation and early organogenesis.

## Introduction

The human genome hosts some 4.5 million sequence inserts readily recognizable as derived from transposable elements (TEs). Most are retroelements, whether ERVs (endogenous retroviruses), LINEs (long interspersed nuclear elements), SINEs (short interspersed nuclear elements, which include primate-specific Alu repeats) or SVAs (SINE-VNTR-Alu, composites of an ERV and Alu, restricted to hominids), which replicate through a copy-and-paste mechanism with reverse transcription of an RNA intermediate followed by insertion of its DNA copy. TEs are increasingly recognized as major drivers of genome evolution owing to their recombinogenic and regulatory potential, even though most are unable to spread further due to inactivating mutations^1,2^.

TEs are tightly controlled by epigenetic silencing mechanisms, yet many are expressed when these mechanisms are put on hold in the context of genome reprograming during gametogenesis or in pre-implantation embryos, when transposition results in inheritable new integrants. Correspondingly, thousands of TEs, albeit mostly primate-restricted ERVs (HERV9, HERVK, HERVL, HERVH), SVAs, Alus, and young LINE-1s, display marks of open chromatin and are transcribed during embryonic genome activation (EGA)^3–6^. The same subset of TEs is enriched in acetylated histone, a hallmark of enhancers, in human embryonic stem cells (hESCs) derived from the pre-implantation embryo^7–9^, where many are bound and controlled by pluripotency transcription factors (TFs)^7,9–13^. This broad induction of transcriptionally active TE loci likely contributes to the efficiency of EGA, and their *cis*-regulatory influences shape the gene regulatory landscape of the pre-implantation embryo. In a remarkable regulatory feedback loop, the pluripotency factor-mediated activation of primate-restricted TE-embedded enhancers leads to the expression of evolutionary contemporaneous Krüppel-associated box (KRAB)-containing zinc finger proteins (KZFPs), which act as sequence-specific repressors of these EGA-induced TEs^9,14^.

KZFP-induced heterochromatin formation and DNA methylation are often viewed as responsible for maintaining TE-embedded regulatory sequences in a repressed state at later stages of development and in adult tissues^15–18^. However, it has become established that TE-embedded regulatory sequences (TEeRS) influence multiple aspects of human or mouse biology, and it is increasingly recognized that KZFPs exert profound influences on their actions^2,16,19–24^. Here, we report that many TEeRS induced during EGA are re-expressed during gastrulation and exhibit an open chromatin in fetal tissues, with a high degree of lineage specificity reflecting their activation no longer by pluripotency factors but by cell-type-restricted TFs. We also determine that many of these TE-derived enhancers are enriched near KZFP genes, the secondary stimulation of which is responsible for lineage-specific heterochromatin formation during germ layer formation. We thus conclude that evolutionary recent TEs and their KZFP controllers strongly influence and thereby confer a high degree of species specificity to the conduct of human gastrulation and fetal development.

## Results

### Cell-type-specific expression of primate-restricted TEs during human gastrulation

**Fig. 1:**
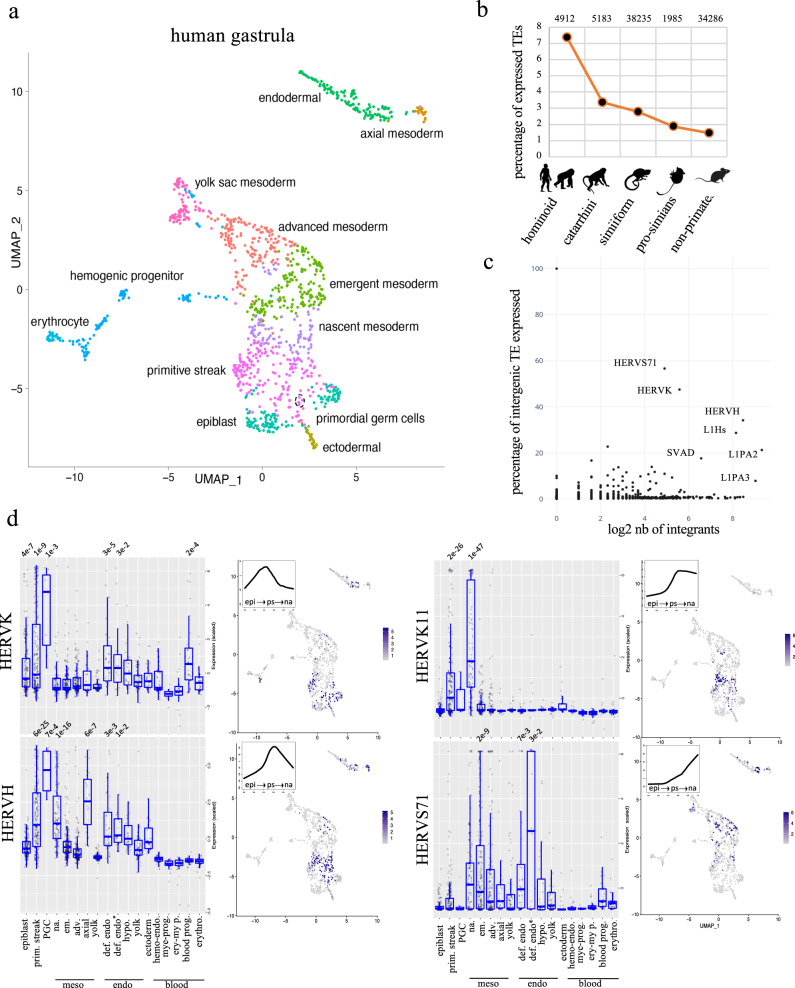
Cell-type-specific expression of primate-restricted TEs during human gastrulation. **a** Cellular composition of the human gastrula. UMAP (Uniform Manifold Approximation and Projection) based on gene expression in single cells from human gastrula; colors correspond to the different cell types identified in ref. 69. **b** Age distribution of TEs expressed in human gastrula. All examined TE subfamilies (excluding DNA transposons) were assigned their evolutionary age category and percentage of expressed integrants from each age category was plotted. On top, the number of expressed integrants are indicated. **c** Relative expression of TE subfamilies in human embryo, depicting number (*x*-axis) and percentage (*y*-axis) of integrants expressed from indicated subfamilies. **d** Cell-type-specific expression of indicated TE subfamilies; each dot represents normalized expression of TE in one cell, grouped in boxplots corresponding to one specific cell type of human gastrula sub-clustering: epiblast (133 cells), primitive streak (prim. streak, 195 cells), primordial germ cells (PGC, 7 cells), nascent mesoderm (na. 98 cells), emergent mesoderm (em. 185 cells), advance mesoderm (adv. 164 cells), axial mesoderm (axial, 23 cells), yolk mesoderm (yolk, 83 cells), definitive endoderm (def. endo. 35 cells), definitive endoderm non-proliferative (def. endo*, 18 cells), hypoblast (hypo, 29 cells), yolk endoderm (yolk, 53 cells), ectoderm (ectoderm 29 cells), hemogenic endothelium (hemo-endo. 37 cells), myeloid progenitor (mye-prog. 17 cells), erythro-myeloid progenitor (ery-my p. 28 cells), blood progenitor (blood prog. 29 cells), erythrocyte (erythro. 32 cells); pseudo-times are indicated in the upper left corner of the UMAPs; time points were extracted from Tyser et al. including epibast (epi), primitive streak (ps) and nascent mesoderm (na) cells; their average expression values are indicated on the *y-axis*, and pseudo-times on the *x*-axis. Significant adjusted *p*-value of expressed TE subfamily in each cell type compared to all others are indicated on top of each boxplot (*p*-value are established using non-parametric Wilcoxon rank sum test).

To examine the transcriptional state of TEs in the immediate post-implantation period, we analyzed single-cell transcriptome data from a gastrulating human embryo^25^. Gene expression patterns allowed the grouping of cells in clusters corresponding to epiblast, primitive streak, primordial germ cells (PGCs), ectoderm, to nascent, emergent, advanced, yolk sac and axial mesoderm, to endoderm and to early hematopoietic compartment (Fig. 1a, Fig. S1a). Overall, we could detect transcripts emanating from more than 100,000 TE loci, with a marked relative overrepresentation of primate-specific integrants (Fig. 1b, Fig. S1b). Many of these evolutionary recent TEs belong to the SVA, HERVK, HERVH, L1PA3, L1PA2, and L1Hs subgroups (Fig. 1c), previously found to be transcribed during EGA^3,9^. However, these EGA-induced subfamilies were more highly expressed in the primitive streak compared to the epiblast, consistent with de novo transcription, with some displaying further cell-specific patterns of expression (Fig. 1d, Fig. S1c, d). For instance, HERVH expression was broad but highest in PGC and axial mesoderm and low in hematogenic derivatives, whereas HERVK transcripts were most abundant in PGCs, endodermal cells, and blood progenitors (Fig. 1d, Fig. S1c, d). Interestingly, the evolutionary young LTR5Hs-HERVK were expressed in both PGCs and endodermal cells, while the more ancient LTR5B-HERVK were detected only in the latter tissue (Fig. 1d, Fig. S1c, d). Moreover, HERVK11 and HERVS71/HERVK22 transcripts were abundant in the primitive streak and nascent mesoderm for the former and in definitive endoderm for the latter, but were not present in epiblast cells or previously detected during EGA (Fig. 1d, Fig. S1c, d). HERVIP10FH and HERV17 integrants were similarly de novo expressed in PGCs (Fig. 1d, Fig. S1c, d).

### Evolutionarily recent TEs exert cell type-specific cis-regulatory influences during human development

**Fig. 2:**
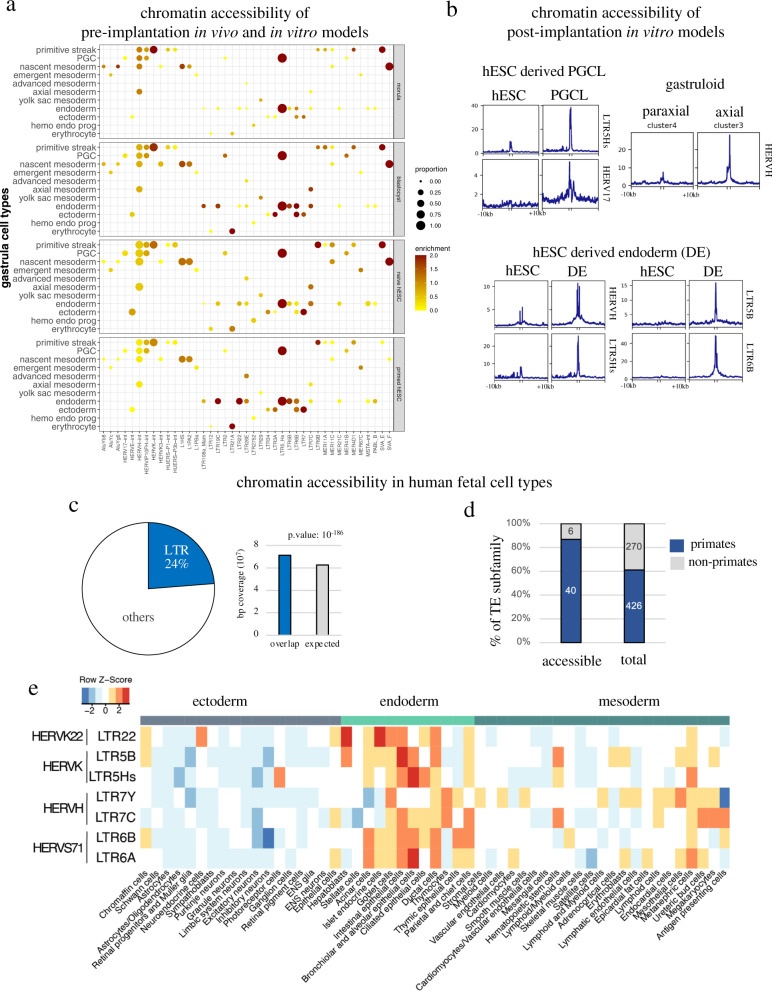
Evolutionarily recent TEs maintain their cis-regulatory potential during human gastrulation and fetal development. **a** Chromatin accessibility of in vivo and in vitro pre-implantation models. The *x*-axis represents the cell type-specific TE subfamilies in human gastrula (*p* < 10e-5, *p*-value are established using non-parametric Wilcoxon rank sum test) that are also accessible in one of the in vivo or in vitro models (*p* < 10e-3). The *y*-axis represents the different models, including chromatin accessibility data from morula/blastocysts^4^ and primed/naïve hESCs; the size of the circles represents the number of accessibility sites overlapping with a specific TE subfamily, normalized by the number of elements in that subfamily; the color intensity represents the log enrichment relative to the random distribution of this overlap. **b** Chromatin accessibility of post-implantation in vitro models. Line plot profiles of chromatin accessibility data at specific TE subfamily in several in vitro models; raw enrichment read average is display + /−10kb around the elements; axial cluster were analyzed from gastruloid perform in this study, EB-derived PGCL and hESC-derived endoderm (DE) and their corresponding hESC were re-analyzed from refs. 26,27. **c** Contribution of LTR TEs to chromatin accessibility at fetal stage. left panel, distribution of all chromatin accessible sites in human fetus (more than 1 million loci) and the proportion overlapping LTR; right panel, measured vs. expected contribution of LTR-derived TE (*p*-value are established using Homer algorithm). **d** Relative accessibility of primate or non-primate TE integrants in human fetus re-analyzed from refs. 4,29,30, indicating number of TE subfamilies with significant accessibility in at least one developmental context (>2 fold enrichment over random genome coverage, *p* < 0.05 established using Homer algorithm). **e** Enrichment over random distribution of selected TE subfamilies in indicated cell types during fetal development re-analyzed from ref. 29; unknown, maternal and placental cell types were removed.

Having documented the germ layer-specific expression of EGA-induced and several other TE subfamilies during human gastrulation, we next asked whether their stage- or lineage-restricted expression correlated with cell type-specific chromatin accessibility during human development. For this, we examined several in vivo and in vitro pre- and early post-implantation model systems. First, we re-analyzed chromatin accessibility dataset obtained from pre-implantation morula and blastocyst^4^ and their respective in vitro derivatives naïve and primed human embryonic stem cells (hESC)^9^, observing that many TEs expressed in specific cells of the gastrula exhibited some level of chromatin accessibility in the pre-implantation embryo and in hESC (Fig. 2a). Interestingly, most accessible subfamilies also have enhancer activity when tested in hESC with an episomal reporter assay^13^ (Supplementary Table 1). Next, we turned to in vitro differentiated hESC derivatives. We re-analyzed single-cell RNA-seq datasets generated from hESC-derived embryoid bodies (EBs) and chromatin accessibility studies performed in their purified primordial germ cells (PGCs) derivatives^26^ (Fig. S2a), as well as transcriptome and chromatin data from hESC-derived endodermal cells^27^. In addition, we experimentally profiled chromatin accessibility and RNA expression at the single nucleus level in gastruloid, a self-organizing elongated embryoid body recently proposed as an in vitro model to study some aspects of human gastrulation^28^ (Fig. S2e–j). We found that in all these systems several TE subfamilies displayed cell-specific expression and chromatin accessibility patterns that matched their expression in the gastrula. For instance, the chromatin of LTR5Hs and HERV17 integrants expressed in PGCs was opened in EB-derived PGCs (Fig. 2b, S2b, d); as well, RNA levels and chromatin accessibility were matched for HERVH integrants in the axial mesoderm and in gastruloid-derived axial cells (Fig. 2b, S2k, l); finally, the endoderm-expressed LTR5B/Hs-HERVK, LTR6B-HERVS71, and HERVH displayed an open chromatin in hESC-derived endoderm (Fig. 2b, S2c). Of note, in some of these cases, chromatin accessibility was noted in hESCs and pre-implantation embryo, but it significantly increases in a TE-subfamily and lineage-specific fashion in the corresponding differentiated derivative.

We then turned to later developmental stages, by comparing single-cell ATAC-seq data generated from 15 organs containing 54 different cell types derived from 12- to 17-week-old human fetuses^29^ with those obtained in pre-implantation embryo^4^ and in vitro-differentiated hESCs^30^. This led us to three observations. First, while ERVs contribute only about 8% of the human genome through some 600,000 integrants, they accounted for a quarter of the 1 million chromatin-accessible loci detected in fetal tissues (Fig. 2c). Second, the most significantly accessible TE subfamilies were largely primate-restricted (Fig. 2d). Third, profiles derived from single-cell chromatin accessibility of endodermal fetal organs revealed differential accessibility for distinct TE subfamilies that corresponded to their germ layer-restricted expression (Fig. 2e).

Together, these results suggest a model whereby chromatin at evolutionarily recent TE loci is opened in the human embryo, with lineage-specific patterns of accessibility and expression in the gastrula influencing the chromatin and transcriptional landscape of later developmental stages.

### Tissue-specific transcription factors control lineage-restricted TE expression during human gastrulation

**Fig. 3:**
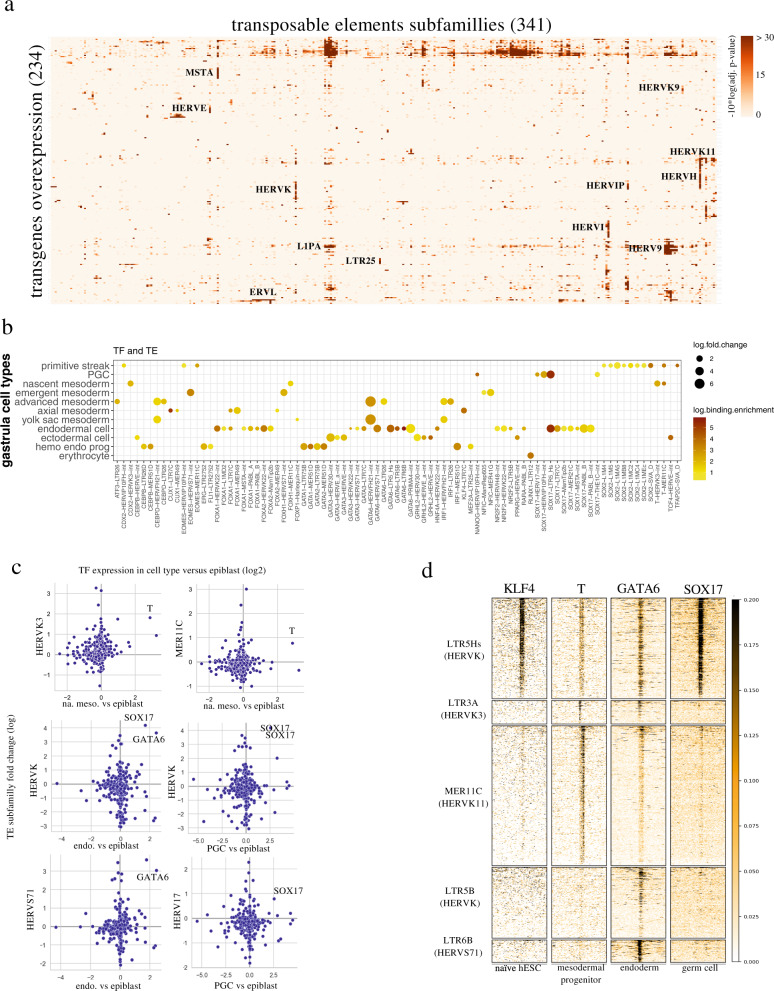
Evolutionary recent TEs act as cell-type-specific enhancers during human gastrulation and fetal development. **a** Enrichment analysis of TE expression induced by transcription factor (TF) overexpression in hESC. Red color intensity corresponds to the enrichment (−10*log10[adjusted *p*-value]) of significantly up-regulated TEs over-representation for a given TE subfamily, re-analyzed from ref. 31; only transgene overexpression conditions and TE subfamilies with an overrepresentation of increased expression (adjusted *p* < 0.05, two-sided *t* test with *p* value correction for multiple testing using the Benjamini–Hochberg’s method) among TEs expressed in each condition at least once are shown. **b** Tissue-specific TEs are induced by tissue-specific TFs. Heatmap of paired tissue-specific TEs and TFs identified in the human gastrula (adjusted *p* < 0.05, two-sided *t* test with *p* value correction for multiple testing using the Benjamini–Hochberg’s method). This list was intersected with the ~2000 TFs and paired TEs identified to bind (adjusted *p* < 0.05) and to induce expression of TE subfamilies (*p* < 0.05); Dot size is proportional to the log of fold change of induction upon overexpression of TFs in hESCs; Color intensity corresponds to the log of binding enrichment. **c** Scatter plot illustrating the coupling between germ layer-specific TFs and TE subfamilies. *y*-axis, TE subfamily log2 fold change expression of intergenic subfamily (excluding all reads overlapping a TE in an exon, an intron and /−10 kb of protein coding gene bodies) induced by overexpressed TF in hESC; *x*-axis, log2 fold change expression of these TFs in human gastrula versus epiblast cells. **d** Binding of transcription factors to their expression-sensitive TE subfamilies. Heat map of the binding profile of transcription factors to all TE elements of the indicated subfamilies. We performed ChIP-seq of KLF4 in naive hESCs; ChIP-seq of GATA6 and SOX17 were respectively re-analyzed from hESC-derived endodermal cells^27^ and a germ cell line^70^. Black intensity reflects the binding strength of the transcription factors.

While the combined expression and accessibility of many TEs in pre-implantation embryo and hESCs reflects their recognition by pluripotency factors (Fig. S3a), the patterns observed at later stages for EGA-induced TE expression suggested regulation by cell type-specific TFs. To probe this hypothesis, we examined the transcriptional changes induced at TEs by individual overexpression of 328 TFs in epiblast-derived human embryonic stem cells (hESC), available through a recent publication^31^ (Fig. S3b, c). We found that, whereas more than 200,000 TE different loci were deregulated in the sum of all these experiments, each TF significantly induced only a restricted set of TE subfamilies (Fig. 3a, Fig. S3d, e). TEs previously noted to be transcribed during the minor and major waves of EGA, such as HERVL and HERVK respectively, were activated by their known cognate activators DUX4 and KLF4 (Fig. 3d, Fig. S3f), supporting the validity of our approach.

By matching the binding profiles of 268 TFs as defined in various cellular contexts^32^ with transcriptome studies performed in overexpressing hESCs^31^, we identified 156 factors that could bind to and induce the expression of 667 TE subfamilies (Supplementary Data 1). We then determined that 92 of these TF-TE pairs were expressed in gastrulating human embryos (Fig. 3b). Among them, overexpression in hESC of TFs considered as markers of particular germ layers, such as Brachyury for early mesoderm, GATA6 for meso-endoderm and SOX17 for endoderm and PGCs, induced the TE subfamilies expressed in the corresponding cells of the gastrulating embryo (Fig. 3b–d). Furthermore, these patterns correlated with their binding specificity, with the meso-endoderm-specific GATA6 recognizing and inducing both LTR5Hs-HERVK and LTR5B-HERVK integrants, but the endoderm/PGC-specific SOX17 doing so only on LTR5Hs-HERVK, as observed in gastrulating embryos (Fig. 3c, d, Fig. S3g).

### Cell-type-specific TEeRS control gene expression during human gastrulation

Since chromatin accessibility is a known marker of *cis*-regulatory elements and reflects TF binding we asked whether the activation of TEeRS by cell-specific TFs controlled the expression of genes situated in their vicinity. To this end, we developed an algorithm aimed at predicting *cis*-regulatory activity^33^. In brief, we used a linear regression model to seek a correlation between the presence of specific TE subfamily members in the proximity of deregulated genes, which we expressed as an enhancer activity prediction score (Fig. S4a). To validate this approach, we inhibited simultaneously SVA- and LTR5Hs-embedded transcriptional units by dCAS9-KRAB (CRISPRi)-mediated repression in naïve hESC. We then calculated the *cis*-regulatory activity prediction scores of TE subfamilies, and confirmed that those most significantly affected in this setting corresponded to SVAs and LTR5Hs HERVK subfamilies targeted by CRISPRi (Fig. S4b). Conversely, KLF4 overexpression in primed hESC resulted in an increased *cis*-regulatory activity prediction score for LTR5Hs-derived HERVK (Fig. S4c), supporting our previous observation that this TF binds to these units and induces their transcription and acquisition of the H3K27ac active chromatin mark^9,31^.

**Fig. 4:**
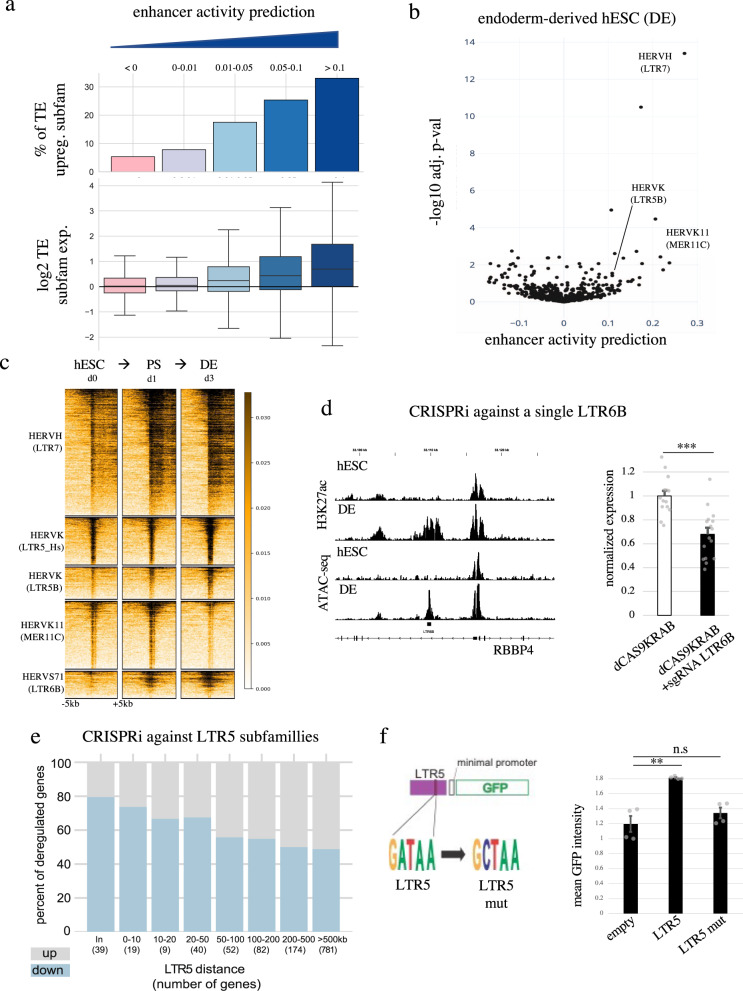
Cell-type-specific TEs control gene expression during gastrulation. **a** Enhancer activity prediction of TE subfamilies correlates with their transcriptional activation. Enhancer activity prediction was established for all TE subfamilies in all overexpressed transgene conditions and grouped based on their activity values from the lowest to the highest (≤0 with 258071 values, 0–0.01 with 29810 values, 0.01–0.05 with 11411 values, 0.05–0.1 with 1687 value, >0.1 with 476 values); bar plots represent the percentage of transcriptionally induced TE subfamilies (adjusted *p* < 0.05, at the subfamily level of intergenic TEs, two-sided *t* test with *p* value correction for multiple testing using the Benjamini–Hochberg’s method) in each category; boxplots represent log2 fold TE subfamily add-up of normalized read count expression change. **b** TE-derived enhancer activity prediction upon endoderm differentiation. Representation of enhancer activity prediction for all TE subfamilies after comparing the transcriptome of hESC and hESC-derived endodermal cells after 3 days of differentiation; *x*-axis represents the activity value and the *y*-axis, the -log10 adjusted p-value (establish by null significance hypothesis testing on the linear regression coefficients and accounted for multiple testing using the Benjamini–Hochberg procedure). **c** H3K27ac enrichment over TE subfamily during hESC-derived endodermal differentiation. Black intensity correlates to H3K27ac ChIP-seq signal + /−10 kbp around all TEs from a named subfamily. **d** *RBBP4* is controlled by an LTR6B endoderm-specific enhancer. Left panel, genome browser of enhancer hallmark landscape (H3K27ac)^71^ and chromatin accessibility profile (ATAC-seq)^27^ of the promoter region of the *RPPB4* gene in hESC and hESC-derived endoderm cells (DE); right panel, normalized *RBBP4* RTqPCR result (over beta-actin and empty) of CRISPRi transduction with (+sgRNA) or without (open) sgRNAs targeting the LTR6B integrant upstream of *RBBP4* followed by endodermal differentiation; error bars indicate SEM and p-value using a two-sided t-test (***: 4e-05) of 14 measurement generated by 4 biologically independent experiments. **e** Impact of LTR5-targeting CRISPRi on gene expression during endodermal differentiation. Number of up- and downregulated genes (*p* < 0.05. two-sided *t* test) at an indicated distance from closest CRISPRi-targeted TE is shown (in: TE within a gene). **f** LTR5 tissue-specific enhancer activity depends on GATA6. Left, schematic representation of the GFP-expressing vector harboring the LTR5B-derived enhancer fragment bound by GATA6 upstream of a minimal promoter; right, GFP activity illustrating the GATA6-dependent enhancer activity of LTR5B; error bars represent SEM and *p*-value using a two-sided *t*-test (**: 0.01, n.s: 0.3) of 4 measurements generated by 2 biologically independent experiments.

We then applied our algorithm to the analysis of the 328 hESC-TF overexpression datasets. We proceeded to rank TE subfamilies according to their *cis*-regulatory activity prediction scores in each condition and found a strong correlation with both the percentage of up-regulated integrants and the overall level of induction of this TE subset for a given TF (Fig. 4a). Importantly, we also calculated the *cis*-regulatory activity prediction score of individual TE subfamilies in hESC-derived endodermal cells and found it to be significant for HERVK11, LTR5B- HERVK and HERVH (Fig. 4b), with our enhancer prediction scores correlating with increased H3K27ac loading, chromatin accessibility and expression of these TE integrants (Fig. 4c, Fig. S4d). To investigate experimentally the regulatory potential of a TEeRS located near a developmentally important gene, we targeted a putative enhancer harbored in an endodermal differentiation-specific TE (LTR6B) located upstream of the PRC2 subunit *RBBP4* with CRISPRi in hESCs, and subjected these cells to an in vitro endodermal differentiation protocol (Fig. 4d, left panel). CRISPRi-induced repression of this LTR6B integrant resulted in *RBBP4* downregulation (Fig. 4d, right panel). Of note, the *RBBP4* gene is more highly expressed in human gastrula endoderm than in its murine counterpart, where this enhancer is absent because LTR6B is a primate-restricted ERV (Fig. S4e, ref. 34). To validate our observation functionally at the subfamily level, we also targeted the LTR5 consensus sequence with CRISPRi in hESC (Fig. S4f) and similarly proceeded to endodermal differentiation. This resulted in the downregulation of hundreds of genes located near the corresponding TE integrants (Fig. 4e). Furthermore, we applied our *cis*-regulatory prediction algorithm in this experimental setting and observed that the most significantly affected TE subfamily corresponded to CRISPRi-targeted LTR5B/Hs-derived HERVK (Fig. S4g). In addition, we were able to verify that the GATA6-binding sequence present in LTR5 conferred responsiveness to an enhancer-GFP reporter system in hESC-derived endoderm (Fig. 4f). Finally, by comparing gene expression in human and mouse endoderm we observed that, amongst TE subfamilies, LTR5Hs was one of the best predictor of human-specific *cis*-regulatory activity (Fig. S4h).

Together, these analyses and experimental data confirm that regulatory sequences hosted by TEs can act as species- and tissue-specific enhancers notably at play during early human development.

### Primate-specific *cis*- and *trans*-regulators partner up to shape gene expression during human gastrulation

**Fig. 5:**
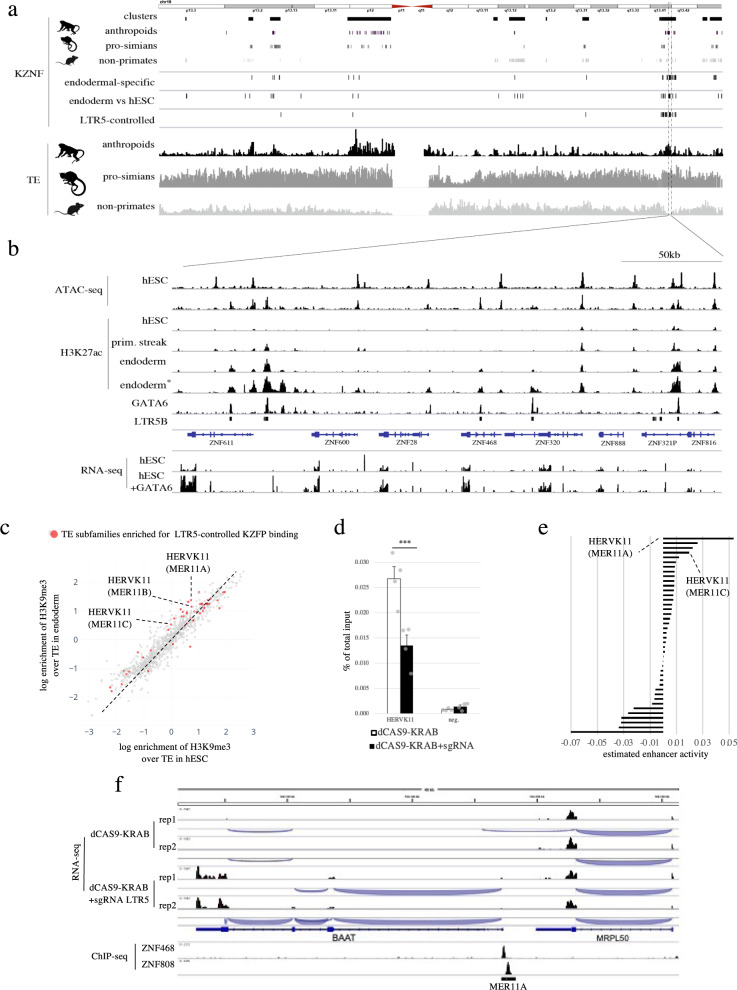
Primate specific *cis*- and *trans*-regulators partner up to control human gastrulation. **a** Transcriptional KZFP regulation during endodermal differentiation, evolutionary age, and genomic distribution of TEs and KZFPs located on chromosome 19. Top, schematic representation of human chromosome 19 with KZFP gene clusters and individual KZFP. Middle, from top to bottom: KZFPs significantly upregulated in endodermal compared to other cells in human gastrula, in hESC-derived endoderm compared to hESC cells, and subjected to LTR5 control (as indicated by downregulation upon LTR5-targeting CRISPRi during endodermal differentiation with a *p* < 0.05, used two-sided *t* test). Bottom, TE density classified by evolutionary ages. **b** Chromatin landscape of endodermal-specific KZFP cluster. Top, ATAC-seq and H3K27ac ChIP-seq profiles during endoderm differentiation at embryonic stem cell stage (hESC, day 0), primitive streak stage (prim. streak, day 1), and endodermal stage (endoderm, day 3) re-analyze from ref. 71; H3K27ac (endoderm*) and GATA6 ChIP-seq correspond to the same kinetic dataset than the ATAC-seq re-analyzed from ref. 27; bottom, RNA-seq performed in doxycycline-inducible GATA6 hESC line with (hESC + GATA6) or without (hESC) doxycycline during 48 h, re-analyzed from ref. 31. **c** H3K9me3 differential enrichment in hESC *vs* hESC-derived endoderm. H3K9me3 TE subfamily enrichment was plotted for all subfamilies containing at least 10 integrants enriched in this mark in at least one condition; in red represent the TE subfamily significantly targeted by LTR5-controlled KZFPs. **d** H3K9me3 loss at HERVK11 upon LTR5-mediated repression during endodermal differentiation. Panel represents H3K9me3 ChIP-qPCR upon LTR5 repression at this same HERVK11 elements; error bars indicate SEM and two-sided *t* test were performed with 0.176 non-significant (n.s) or significant (***) 0.006 *p*-value results on biological quadruplicates. **e** *Cis*-regulatory estimation of TE subfamily bound by LTR5-controlled KZFPs in hESC-derived foregut with or without LTR5 mediated repression. **f** Transcriptional landscape of MER11A controlling BAAT expression in hESC-derived foregut with or without LTR5 mediated repression; bottom lanes are ChIP-seq of KZFP from refs. 72.

Of the hundred nearby genes downregulated upon LTR5 repression (Fig. S4i), 21 encode for KZFPs, a finding of interest since many members of this large family of DNA-binding proteins are responsible for silencing TEs through H3K9me3 deposition, histone deacetylation and DNA methylation^35^. Because of their expansion by gene and segment duplications, many *KZFP* genes are grouped in clusters, notably on human chromosome 19. We observed that *KZFP* genes located in the same genomic cluster are often of similar evolutionary age and are generally surrounded by insertions of contemporaneous TE subfamilies (Fig. 5a, Fig. S5a, b). Moreover, we noted that while *KZFP* gene expression globally decreases during differentiation, primate-restricted clusters display coordinated upregulation in specific cells of human gastrula (Fig. S5c, d).

Most *KZFP* genes (19 genes, 17 of them primate-restricted) repressed upon LTR5 CRISPRi-mediated silencing during endodermal differentiation reside in one such cluster, which we found to be enriched in LTR5B inserts (Fig. 5a). Eleven of these *KZFP* genes, which include *ZNF611*, *ZNF600*, *ZNF28*, *ZNF468* and *ZNF320*, were more expressed in human gastrula endodermal cells and during in vitro endodermal differentiation of hESCs (Fig. 5ab). Correspondingly, we determined that GATA6 bound directly to numerous LTR5B integrants during endodermal differentiation and induced expression of LTR5B-HERVK and nearby *KZFP* genes when overexpressed in hESC (Fig. 5b). Additionally, CRISPRa-mediated activation of LTR5 in NCCIT teratocarcinoma cells led to the induction of *KZFP* genes flanked by integrants belonging to this TE subset (Fig. S5e, ref. 36).

Interestingly, we observed strong changes in the H3K9me3 landscape of TE loci between hESC and hESC-derived endodermal cells and noted a correlation with overlapping targets of LTR5-controlled *KZFP* genes (Fig. 5c). We notably observed LTR5-dependent increases in H3K9me3 enrichment over the transiently expressed HERVK11 subfamily in primitive-streak/nascent mesoderm during in vitro endodermal differentiation (Fig. 5d). Furthermore, applying our *cis*-regulatory activity prediction algorithm in the setting of LTR5-repressed endoderm-foregut differentiation yielded the highest score for MER11-HERVK11 integrants. Accordingly, upon CRISPRi-mediated targeting of LTR5 in hESC-derived foregut, we detected activation of several genes bearing MER11 inserts in their vicinity, including IQCG, DYSF and BAAT (Fig. S5f). These three genes were previously identified as co-expressed with MER11A in liver tissue^37^, and the liver-specific *BAAT*, mutations of which are associated with familial hypercholanemia^38^, uses a MER11A LTR as its primary promoter^39^. Most interestingly, we previously determined that the MER11A *BAAT* promoter is bound by the LTR5-stimulated endoderm-specific KZFPs ZNF468 and ZNF808 (Fig. 5f). This suggests a regulatory cascade where GATA6-mediated activation of LTR5 induces the expression of KZFPs repressing the transcription of MER11A-controlled genes in differentiating endoderm.

More generally, our results suggest a model whereby master TFs trigger a chain reaction during gastrulation by activating primate-restricted TEs controlling KZFPs of similar evolutionary age, which in turn repress these and other TE-based *cis*-acting regulatory elements, contributing to shape the chromatin and transcriptional landscape of the different germ layers. Therefore, transcriptional networks at play human development are controlled by a triangular relationship between canonical TFs, their primate-restricted TEeRS and evolutionarily related KZFPs countering their influences, the latter two endowing the regulome of human gastrulation and subsequent steps of fetal development with a high level of species-specificity.

## Discussion

Together, these data demonstrate that transcriptional networks during human early development, while orchestrated by canonical TFs, are shaped by a partnership between primate-restricted TEeRS targeted by these activators and KZFP repressors countering their influences. As such, the regulome of human gastrulation displays a remarkable level of species-specificity reflecting the presence of both recently acquired TE integrants and KZFPs selected to tame their activity.

The broad expression of TEs such as HERVKs, HERVHs, SVAs, and young LINE-1s during EGA and in the PGC lineage^3,40^ is explained by their recognition by stem cell factors expressed during these periods, such as SOX2, OCT4, NANOG or KLF4/17^7,9–13^. New TE integrants must seed the genome during the pre-implantation period or germline formation to become inherited, hence their expression at these timepoints. Interestingly, even though HERVH has been suggested to represent a human embryonic stem cell marker^41^, we observed that HERVH integrants displayed strong accessibility and expression in other cell types during development including PGC, axial mesoderm and definitive endoderm.

Our data indicate that TEeRS regulate subsequent steps of embryonic development in part through the recruitment of TFs active in germ layer determination, such as GATA6 and SOX17. More generally, it is well established that TEs can harbor binding sites for a wide array of TFs active in differentiated tissues, and the expression of some somatic genes, for instance in the immune system, is driven by TEeRS^42–45^. Additionally, primate-specific TEeRS are major contributors to *cis*-regulatory innovations in hESCs and adult liver^46,47^. The *cis*-regulatory influences of these recently emerged TEeRS on human fetal development suggest that, in spite of the evolutionary constraints proposed by the hourglasses developmental model^48^, all stages of this process are subject to regulatory innovation, and it is likely that gastrulation is influenced by lineage-restricted TE-hosted enhancers in other species as well, as already noted for placentation^49^. Supporting this hypothesis, tissue-specific TE subfamily expression was recently observed during mouse gastrulation^50^.

How the presence of binding sites for TFs typically active in differentiated tissues, which is predicted to promote neither the spread nor the inheritability of TEs, came to be selected through evolution is the object of much speculation^51,52^. However, we note here that primate-restricted ERVs are vastly overrepresented amongst TEs that harbor somatic TF binding sites, are expressed during gastrulation, and display an open chromatin state during fetal development. This is consistent with the observation that many act as enhancers or promoters in developing or differentiated organs^53^. As ERVs are derived from exogenous retroviruses that once replicated in somatic tissues and were largely endowed with oncogenic properties favoring their expansion and persistence, it maybe that the diversity of TF binding sites harbored by ERVs is just a consequence of their ancestry. But how was this feature maintained in evolution? Our finding that TEs activated during either EGA or gastrulation stimulate the transcription of *KZFP* genes, the products of which in turn repress these TEs and confer germ layer-specificity to their transcriptional influences, strongly suggests that KZFPs, rather than just the host side of an evolutionary arms race^54^, were instrumental in allowing for the preservation and exploitation of the broad regulatory potential of TEs in higher vertebrates.

## Methods

### hESC culture and differentiation

H9 and H1 human ESC line were maintained in mTSER plus on Matrigel and were passaged using TryplE in single cells. Endodermal differentiation was performed as in ref. 27. Briefly, hESC were passaged at 80 k cells/cm2 density in 12-well Matrigel-coated plate. When cells reached 80–90% confluence at 48–76 h post-splitting, endodermal differentiation was initiated with 100 ng/ml of Activin A for 3 days, 5 μM GSK-3 inhibitor (CHIR-99021) to activate the WNT pathway for the first day, and 0.5 μM for the second day. Pancreatic differentiation was performed accordingly to a Stem Cell Technology^TM^ protocol (Catalog #05120) with harvest after 3 days of endodermal differentiation followed by 3 days of foregut differentiation. Gastruloid differentiation was performed as in ref. 28. Briefly, hESC were passaged at 40,000 cells/cm^2^ in mTSER replaced 48–76 h post-splitting by Nutristem media with 3 μM CHIR-99021. After 24 h cells were split in single-cell passaging with TryplE, embryoid bodies were formed with 800 cells per well of low adherence 96-well plate Cell Star in E6 media supplemented with 3 μM CHIR-99021 and 10 μM Rock inhibitor for 18 h, then replaced with E6 twice to be harvested at 72 h.

### CRISPRi experiments

sgRNA design was performed by taking the Dfam consensus of LTR5B common sequence (TTGCAGTTGAGATAAGAGGAAGG). Furthermore, sgRNA design for LTR6B (GGCTTTGGGCGTTTATCAAT, TTGATAAACGCCCAAAGCCC, TATTACAAGGTGATAGATCC) perform to uniquely match chr1:33109510-33110065 (hg19). Specificity was predicted with the CRISPOR software v5.01^55^. hESCs in H9 media were transduced with dCAS9-KRAB lentiviral vector, selected, and maintained in puromycin (0.25 μg/mL) for 5 to 10 days before differentiation experiments.

### ChIP-qPCR and RT-qPCR

ChIP were performed as in ref. 9 and primer use for HERVK11 were (Fw CCTTCCCATACTCGCAGTTC, Rv TGCATACAAGGACCAGCTCA) and for Neg (CCAATTTCGTGCCTCATTTT. TCAGCATGTCTCCTTTGCTG), RBBP4 (Fw ATGACCCATGCTCTGGAGTG, Rv GGACAAGTCGATGAATGCTGAAA) gene expression were normalized with ACTB (Fw CATGTACGTTGCTATCCAGGC, Rv CTCCTTAATGTCACGCACGAT). Reverse Transcriptions were performed using Thermo Scientific Maxima™ H Minus cDNA SynthesisMaster Mix ref #M1661.

### ChIP-seq

Sequenced reads were aligned to the reference human genome hg19 with bowtie2^56^. MACS 2.2.4^57^ was used for peak calling. Peaks were merged using bedtools v2.27.1^58^. FeatureCounts^59^ was used to count uniquely mapped reads (MAPQ > 10) on the peaks. Samtools tools v1.1 was used to convert in bam files. Library size correction was performed using the TMM method as implemented in the limma package of R, using the total number of aligned reads as size factor. All ChIP-seq binding locations from the literature were extracted from ChIP-Atlas database^32^ containing data for more than a thousand of chromatin associated proteins including more than 15'000 different datasets. Differential analysis on the uniquely mapped counts between conditions was performed with voom^60^. Heatmaps and profile averages were calculated using deeptools v3.5.1^61^ over 5 kb windows around the peak/repeat center from bigwigs. Enrichment analysis over TE subfamilies was performed with HOMER software v4.10.4^62^ and visualized in IGV v2.8.4 and ggplots v3.l.1 in R v4.l.2.

### RNA-seq

Total RNA from cell lines was isolated with NucleoSpin™ RNA Plus kit (Machery-Nagel). cDNA was prepared with Maxima Reverse Transcriptase (Thermo Scientific). Sequencing libraries were performed with Illumina Truseq Stranded mRNA LT kit. Reads were mapped to the human (hg19) genome using Hisat2 v2.l.0^63^. Counts on genes and TEs were generated using featureCounts v1.6.2^58^ and only uniquely mapped reads with MAPQ > 10 were kept. To avoid read assignation ambiguity between genes and TEs, a gtf file containing both, genes and TEs was provided to featureCounts. For repetitive sequences, an in-house curated version of the Repbase database was used (fragmented LTR and internal segments belonging to a single integrant were merged). Only uniquely mapped reads were used for counting on genes and repetitive sequences integrants. TEs overlapping exons or that did not have at least one sample with 3 reads were discarded from the analysis. Normalization for sequencing depth was done for both genes and TEs using the TMM method as implemented in the limma package of Bioconductor^64^, with the counts on genes as library size. Finally, for each transgene, differential gene expression analysis was performed using Voom^60^ as it has been implemented in the limma package of Bioconductor v3.13 and assessing only genes (or TEs) that had 3 reads in at least one sample. A gene (or TE) was considered differentially expressed when the fold change between groups was bigger than 2 and the *p*-value was smaller than 0.05. A moderated t-test (as implemented in the limma package of R) was used to test significance. *P*-values were corrected for multiple testing using the Benjamini–Hochberg’s method. For counting on TE subfamilies, we added up reads on repetitive sequences without filtering out for multi-mapped reads and added them up per subfamily.

### Cis-regulatory activity estimation

To identify TE subfamilies exerting putative cis-regulatory activities directly from RNA-seq data, we modeled treatment vs control deviations in gene expression of protein coding genes as a linear combination of occurrences of nearby (within 50 kb of the TSS of protein coding genes) TE subfamily integrants^33^. We excluded TEs overlapping exons and TE subfamilies that colocalized less than 150 times near the promoters of protein coding genes. The coefficients of this linear regression problem can be interpreted as the deviations of logged gene expression values explained by the presence of one TE for each TE subfamily. We define these coefficients as the cis-regulator activity of TE subfamilies. Similar models were proposed to infer the activity of DNA motifs from gene expression data^65^. To find TE subfamilies with significant cis-regulatory activities, we performed null significance hypothesis testing on the linear regression coefficients and accounted for multiple testing using the Benjamini–Hochberg procedure.

### Enhancer reporter system

We used a lentiviral vector containing a minimal promoter followed of GFP cDNA (FpG5, Addgene #69443) containing a full LTR from LTR5 subfamily amplified from a LTR5B (chr19:53226814-53227799, hg19). Then a single mutation was generated using Agilent Technologies QuikChange II XL (Cat#200522-5). H9 hESC were transduced by either of these enhancer-containing vectors followed by 3 days of endodermal differentiation and then analyze by FACS (FlowJo LCC v8.8.7).

### ATAC-seq studies

Reads were aligned with bowtie2. Mitochondrial reads were removed before peak calling. Peak calling was done with MACS2 with *q* < 10^e-5^, and using the --bampe option when PE reads.

### Single-cell multi-omics

Cellranger-arc^66^ was used to obtain counts on genes and peaks using default parameters. The hg38 reference genome provided by cellranger-arc was used. We identified five main clusters in this experimental system: TBXT-expressing axial mesoderm, SOX2-expressing neuro-mesenchymal progenitors (NMP), and three different stages of paraxial mesoderm differentiation preferentially expressing TBX6 (somitic), or PAX3/TWIST1/SIX1 (early paraxial and pre-somitic) with a different degree of somitic HOX gene markers (Fig. 2c, Fig. S2b–e). Cellular clustering matched increased chromatin accessibility with the corresponding cell-type-specific TF binding sites, such as illustrated for TBXT **(**Fig. S2f).

### Single-cell RNA-seq analyses

For the human embryo dataset, counts were obtained using cellranger^67^ using a GTF of hg19 that contained both, genes and TEs. Only uniquely mapped reads on genes that were expressed in at least 1% of the samples and in a minimum 3 cells for TEs were kept. Then, for TEs not overlapping exons, counts were added up at subfamily level. Cells with less than 200 features and more than 25% of mitochondrial reads were removed. Seurat’s SCTransform^68^ was used to normalize the data and correct for mitochondrial percentage and total number of reads biases. For embryoid-body time course differentiation, same method was apply except that only cells with more than 20% of mitochondrial reads were removed. UMAP were computed with Seurat’s R package v4.1.0 with default parameters using the first 30 principal components as input. Of note, our and Tyler et al.’s UMAPs only present minimal differences, yet axial mesoderm and endoderm appear slightly closer when depicted with our analytical pipeline.

### Single-cell ATAC-seq analyses

Single-cell ATAC-seq peaks data were downloaded from the Atlas Of Chromatin Accessibility During Development (https://atlas.brotmanbaty.org/bbi/human-chromatin-during-development/)^29^. Significance for TE family chromatin accessibility enrichment was assessed by random permutations: First, for each TE subfamily, the total number of detected peaks for a given cell type on the selected TE subfamily was computed using R GenomicRanges library v1.48.0. Then, TE subfamilies were randomly shuffled 10 times using bedtools with options –chrom and –noOverlapping and the total number of peaks for each permutation was computed. The fold enrichment of the significant subfamilies was then plotted on a heatmap using R heatmap2 function for each cell type.

### TE density profiling

TE bigwig densities were computed using python. First, TEs, were extracted from the TE database depending on their subfamily evolutionary ages in bed format and converted to bedgraph using the genomecov command of BedTools v2.27.1. Then, bedgraph signals were smoothed using a rectangular window of 10 kb and written in bigwig format using the pyBigWig python library v0.3.18.

### Statistics & reproducibility

No statistical method was used to predetermine sample size. No data were excluded from the analyses. The experiments were not randomized The Investigators were partly blinded to allocation during experiments and outcome assessment. All experiments contain at least 2 biologically independent replicates (for RNA-seq) and more than 3 for ChIP/RT-qPCR and FACS analysis. Figures 1d, S1d, 2a: *p*-value using non-parametric Wilcoxon rank sum test with Seurat algorithm. Figure 2a, c, d, S2c, S3a, S5b, Supp Data 1: *p*-value using Homer algorithm (annotatePeaks.pl). Figures S2c, 4a, 5a, Supplementary Data 1: *p*-value using a two-sided *t* test. Fig. 3a, b, S3d–f, 4a, S5f, Fig. 4b, S4f, g: *p*-value using two-sided *t* test with correction for multiple testing using the Benjamini–Hochberg’s method.

### Reporting summary

Further information on research design is available in the Nature Portfolio Reporting Summary linked to this article.

## Supplementary information

Supplementary Information

Description of Additional Supplementary Files

Supplementary Data 1

Reporting Summary

## Data Availability

The Single-cell multi-omics of gastruloid, RNA-seq of endodermal differentiated hESC with or without LTR5-targeting sgRNA generated have been deposited in the deposited in the Gene Expression Omnibus (GEO) database under accession number GSE181120. without restricted access. All other genomic data of this study were extracted from the GEO database: GSE140021 for the 10X single-cell RNA-seq of hESC-derived embryoid body time course; GSE120648 for ATAC-seq of purified PGC during hESC-derived embryoid body differentiation; GSE117136 and GSE52657 for ChIP-seq and ATAC-seq from endodermal differentiation; GSE130418 for ATAC-seq of naïve and primed hESCs; Single-cell ATAC-seq from fetal organ: https://descartes.brotmanbaty.org/. E-MTAB-9388 for the SMART-seq single-cell RNA-seq of human gastrula from EBI database. DRA006296 for the overexpression dataset of TF in hESC from DDBJ database. hg19 reference genome wer used from UCSC. No restriction for dataset availability. Source data are provided as a Source Data file. Source data are provided with this paper.

## Source data

Source Data

## Code availability

All code used will be provided upon request and is available on Github [https://github.com/julienpontis/TE-Gastrulation].

## References

[1] Sundaram V, Wysocka J (2020). Transposable elements as a potent source of diverse cis-regulatory sequences in mammalian genomes. Philos. Trans. R. Soc. Lond. B Biol. Sci..

[2] Chuong EB, Elde NC, Feschotte C (2017). Regulatory activities of transposable elements: from conflicts to benefits. Nat. Rev. Genet..

[3] Göke J (2015). Dynamic transcription of distinct classes of endogenous retroviral elements marks specific populations of early human embryonic cells. Cell Stem Cell.

[4] Gao L (2018). Chromatin accessibility landscape in human early embryos and its association with evolution. Cell.

[5] Liu L (2019). An integrated chromatin accessibility and transcriptome landscape of human pre-implantation embryos. Nat. Commun..

[6] Wu J (2018). Chromatin analysis in human early development reveals epigenetic transition during ZGA. Nat. Publ. Group.

[7] Kunarso G (2010). Transposable elements have rewired the core regulatory network of human embryonic stem cells. Nat. Genet..

[8] Fort A (2014). Deep transcriptome profiling of mammalian stem cells supports a regulatory role for retrotransposons in pluripotency maintenance. Nat. Genet..

[9] Pontis J (2019). Hominoid-specific transposable elements and KZFPs facilitate human embryonic genome activation and control transcription in naive human ESCs. Cell Stem Cell.

[10] Wang J (2014). Primate-specific endogenous retrovirus-driven transcription defines naive-like stem cells. Nat. Publ. Group.

[11] Ohnuki M (2014). Dynamic regulation of human endogenous retroviruses mediates factor-induced reprogramming and differentiation potential. Proc. Natl Acad. Sci. USA.

[12] Grow EJ (2015). Intrinsic retroviral reactivation in human preimplantation embryos and pluripotent cells. Nature.

[13] Barakat TS (2018). Functional dissection of the enhancer repertoire in human embryonic stem cells. Stem Cell.

[14] Haring NL (2021). ZNF91 deletion in human embryonic stem cells leads to ectopic activation of SVA retrotransposons and up-regulation of KRAB zinc finger gene clusters. Genome Res..

[15] Greenberg MVC, Bourc’his D (2019). The diverse roles of DNA methylation in mammalian development and disease. Nat. Rev. Mol. Cell Biol..

[16] Friedli M, Trono D (2015). The developmental control of transposable elements and the evolution of higher species. Annu. Rev. Cell Dev. Biol..

[17] Guo H (2014). The DNA methylation landscape of human early embryos. Nat. Publ. Group.

[18] Smith ZD (2014). DNA methylation dynamics of the human preimplantation embryo. Nat. Publ. Group.

[19] Fueyo, R., Judd, J., Feschotte, C. & Wysocka, J. Roles of transposable elements in the regulation of mammalian transcription. *Nat. Rev. Mol. Cell Biol.*10.1038/s41580-022-00457-y (2022). 10.1038/s41580-022-00457-y PMC10470143 35228718

[20] Yang, P., Wang, Y. & Macfarlan, T. S. The role of KRAB-ZFPs in transposable element repression and mammalian evolution. *Trends Genet.* 1–11 10.1016/j.tig.2017.08.006 (2017). 10.1016/j.tig.2017.08.006 PMC5659910 28935117

[21] Turelli P (2020). Primate-restricted KRAB zinc finger proteins and target retrotransposons control gene expression in human neurons. Sci. Adv..

[22] Ecco G (2016). Transposable elements and their KRAB-ZFP controllers regulate gene expression in adult tissues. Dev. Cell.

[23] Playfoot CJ (2021). Transposable elements and their KZFP controllers are drivers of transcriptional innovation in the developing human brain. Genome Res..

[24] Wolf G (2015). The KRAB zinc finger protein ZFP809 is required to initiate epigenetic silencing of endogenous retroviruses. Genes Dev..

[25] Tyser RCV (2021). Single-cell transcriptomic characterization of a gastrulating human embryo. Nat. Publ. Group.

[26] Chen D (2019). Human Primordial Germ Cells Are Specified from Lineage-Primed Progenitors. Cell Rep..

[27] Lee K (2019). FOXA2 is required for enhancer priming during pancreatic differentiation. Cell Rep..

[28] Moris N (2020). An in vitro model of early anteroposterior organization during human development. Nat. Publ. Group.

[29] Domcke, S. et al. A human cell atlas of fetal chromatin accessibility. *Science* **370**, eaba7612 (2020). 10.1126/science.aba7612 PMC7785298 33184180

[30] Pastor WA (2018). TFAP2C regulates transcription in human naive pluripotency by opening enhancers. Nat. Cell Biol..

[31] Nakatake Y (2020). Generation and profiling of 2135 human ESC lines for the systematic analyses of cell states perturbed by inducing single transcription factors. Cell Rep..

[32] Oki, S. et al. ChIP-Atlas: a data-mining suite powered by full integration of public ChIP-seq data. *EMBO Rep*. **19**, e46255 (2018). 10.15252/embr.201846255 PMC6280645 30413482

[33] Pulver, C. et al. Statistical learning quantifies transposable element-mediated cis-regulation. *bioRxiv*10.1101/2022.09.23.509180 (2022). 10.1186/s13059-023-03085-7 PMC10637000 37950299

[34] Pijuan-Sala B (2019). A single-cell molecular map of mouse gastrulation and early organogenesis. Nat. Publ. Group.

[35] Ecco G, Imbeault M, Trono D (2017). KRAB zinc finger proteins. Development.

[36] Fuentes, D. R., Swigut, T. & Wysocka, J. Systematic perturbation of retroviral LTRs reveals widespread long-range effects on human gene regulation. *Elife* **7**, e35989 (2018). 10.7554/eLife.35989 PMC6158008 30070637

[37] Pavlicev M, Hiratsuka K, Swaggart KA, Dunn C, Muglia L (2015). Detecting endogenous retrovirus-driven tissue-specific gene transcription. Genome Biol. Evol..

[38] Carlton VEH (2003). Complex inheritance of familial hypercholanemia with associated mutations in TJP2 and BAAT. Nat. Genet..

[39] Cohen CJ, Lock WM, Mager DL (2009). Endogenous retroviral LTRs as promoters for human genes: a critical assessment. Gene.

[40] Tang WWC (2015). A unique gene regulatory network resets the human germline epigenome for development. Cell.

[41] Santoni FA, Guerra J, Luban J (2012). HERV-H RNA is abundant in human embryonic stem cells and a precise marker for pluripotency. Retrovirology.

[42] Bourque G (2008). Evolution of the mammalian transcription factor binding repertoire via transposable elements. Genome Res..

[43] Sundaram V (2014). Widespread contribution of transposable elements to the innovation of gene regulatory networks. Genome Res..

[44] Ito J (2017). Systematic identification and characterization of regulatory elements derived from human endogenous retroviruses. PLoS Genet..

[45] Chuong EB, Elde NC, Feschotte C (2016). Regulatory evolution of innate immunity through co-option of endogenous retroviruses. Science.

[46] Trizzino M (2017). Transposable elements are the primary source of novelty in primate gene regulation. Genome Res..

[47] Jacques P-É, Jeyakani J, Bourque G (2013). The majority of primate-specific regulatory sequences are derived from transposable elements. PLoS Genet..

[48] Development, D. D. Temporal colinearity and the phylotypic progression: a basis for the stability of a vertebrate Bauplan and the evolution of morphologies through heterochrony. journals.biologists.com (1994). 7579514

[49] Sun M-A (2021). Endogenous retroviruses drive lineage-specific regulatory evolution across primate and rodent placentae. Mol. Biol. Evolution.

[50] He J (2021). Identifying transposable element expression dynamics and heterogeneity during development at the single-cell level with a processing pipeline scTE. Nat. Commun..

[51] Sundaram, V. & Wang, T. Transposable element mediated innovation in gene regulatory landscapes of cells: re-visiting the ‘Gene-Battery’ model. *BioEssays* **40**, (2018). 10.1002/bies.201700155 PMC5912915 29206283

[52] Britten RJ, Davidson EH (1971). Repetitive and non-repetitive DNA sequences and a speculation on the origins of evolutionary novelty. Q Rev. Biol..

[53] Pehrsson EC, Choudhary MNK, Sundaram V, Wang T (2019). The epigenomic landscape of transposable elements across normal human development and anatomy. Nat. Commun..

[54] Jacobs FMJ (2014). An evolutionary arms race between KRAB zinc-finger genes ZNF91/93 and SVA/L1 retrotransposons. Nature.

[55] Haeussler M (2016). Evaluation of off-target and on-target scoring algorithms and integration into the guide RNA selection tool CRISPOR. Genome Biol..

[56] Langmead B, Salzberg SL (2012). Fast gapped-read alignment with Bowtie 2. Nat. Methods.

[57] Zhang Y (2008). Model-based analysis of ChIP-Seq (MACS). Genome Biol..

[58] Quinlan AR, Hall IM (2010). BEDTools: a flexible suite of utilities for comparing genomic features. Bioinformatics.

[59] Liao Y, Smyth GK, Shi W (2014). featureCounts: an efficient general purpose program for assigning sequence reads to genomic features. Bioinformatics.

[60] Law CW, Chen Y, Shi W, Smyth GK (2014). voom: Precision weights unlock linear model analysis tools for RNA-seq read counts. Genome Biol..

[61] Ramírez F, Dündar F, Diehl S, Grüning BA, Manke T (2014). deepTools: a flexible platform for exploring deep-sequencing data. Nucleic Acids Res..

[62] Heinz S (2010). Simple combinations of lineage-determining transcription factors prime cis-regulatory elements required for macrophage and B cell identities. Mol. Cell.

[63] Kim D, Langmead B, Salzberg SL (2015). HISAT: a fast spliced aligner with low memory requirements. Nat. Methods.

[64] Gentleman RC (2004). Bioconductor: open software development for computational biology and bioinformatics. Genome Biol..

[65] Balwierz PJ (2014). ISMARA: automated modeling of genomic signals as a democracy of regulatory motifs. Genome Res..

[66] Satpathy AT (2019). Massively parallel single-cell chromatin landscapes of human immune cell development and intratumoral T cell exhaustion. Nat. Biotechnol..

[67] Molè MA (2021). A single cell characterisation of human embryogenesis identifies pluripotency transitions and putative anterior hypoblast centre. Nat. Commun..

[68] Stuart T (2019). Comprehensive integration of single-cell data. Cell.

[69] Tyser, R. C. V. et al. A spatially resolved single cell atlas of human gastrulation. *bioRxiv* 2020.07.21.213512 10.1101/2020.07.21.213512 (2020).

[70] Jostes SV (2020). Unique and redundant roles of SOX2 and SOX17 in regulating the germ cell tumor fate. Int. J. Cancer.

[71] Loh KM (2014). Efficient endoderm induction from human pluripotent stem cells by logically directing signals controlling lineage bifurcations. Cell Stem Cell.

[72] Imbeault M, Helleboid P-Y, Trono D (2017). KRAB zinc-finger proteins contribute to the evolution of gene regulatory networks. Nat. Publ. Group.

