# Supplementary material for: Primate-specific transposable elements shape transcriptional networks during human development
Source: Nat Commun. 2022 Nov 23;13:7178. doi: 10.1038/s41467-022-34800-w (PMC9684439; doi:10.1038/s41467-022-34800-w)
Supplement: Supplementary file 1 — Supplementary Information [file 41467_2022_34800_MOESM1_ESM.pdf]

Supplementary Information

Primate-specific transposable elements shape transcriptional networks during human development

Pontis et al.

Fig. S1: Cell-type Specific Expression of Primates TEs during Human Gastrulation.

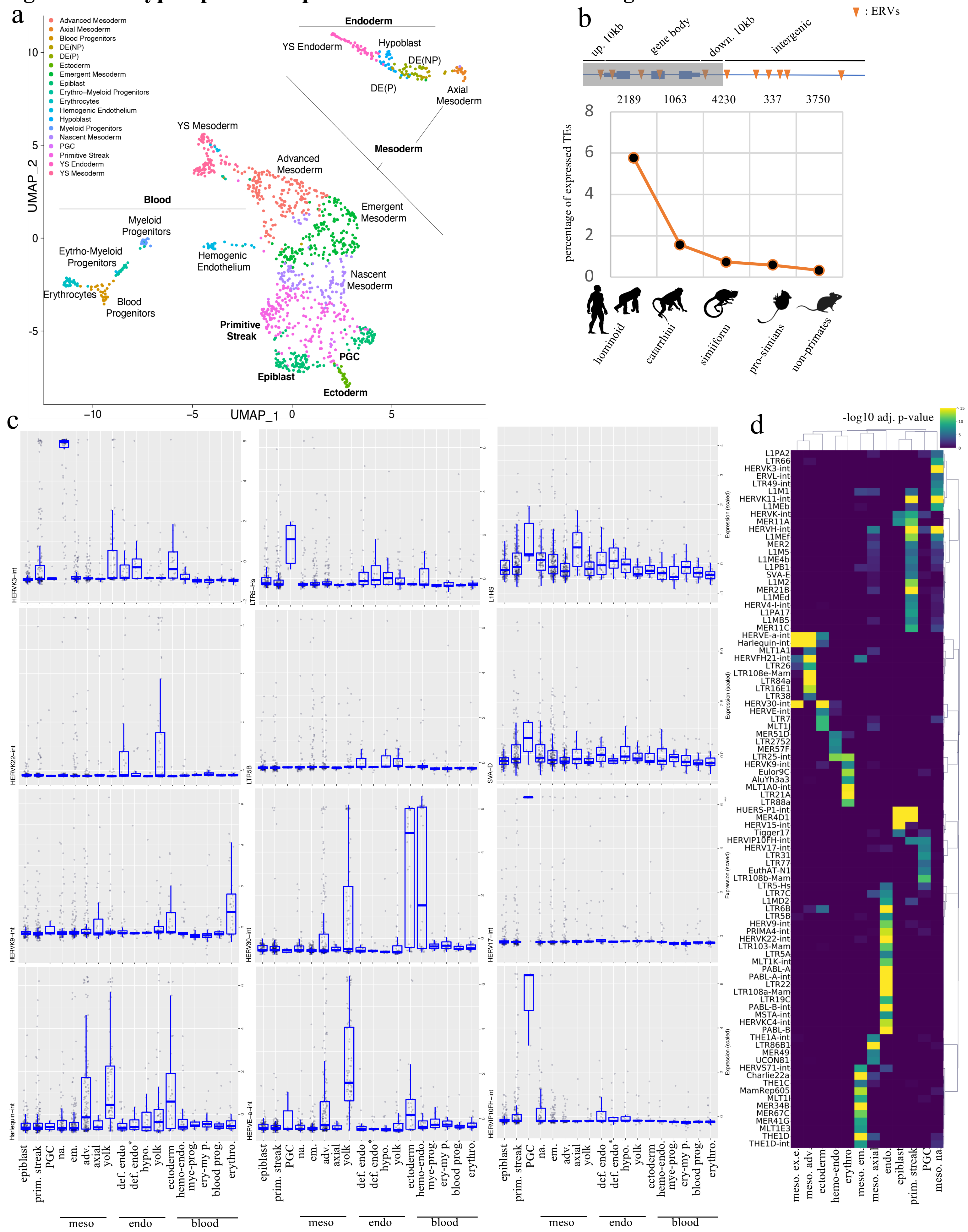

Figure S1 for Fig 1. ***Cell-type-specific expression of primate-restricted TEs during human gastrulation.***

a, Cellular composition of human gastrula. UMAP based on single-cell gene expression from human gastrula. Colors represent more detailed cell subtypes identified in[67]. **b**, Age distribution of expressed intergenic TEs in human gastrula. Each TE subfamily (excluding DNA transposons) was restricted to a specific evolutionary age category, and the percentage of expressed TE integrants in each was plotted. We excluded any TEs overlapping coding gene body and up/down-stream of a gene, thus removing TEs expressed due to readthrough or gene transcript inclusion. **c,** Cell-type-specific expression of TE subfamilies; boxplots with each dot representing the TE subfamily normalized expression in one cell. Cells were grouped in boxplots corresponding to one cell type of human gastrula sub-clustering: epiblast (133 cells), primitive streak (prim. streak, 195 cells), primordial germ cells (PGC, 7 cells), nascent mesoderm (na. 98 cells), emergent mesoderm (em. 185 cells), advance mesoderm (adv. 164 cells), axial mesoderm (axial, 23 cells), yolk mesoderm (yolk, 83 cells), definitive endoderm (def. endo. 35 cells), definitive endoderm non-proliferative (def. endo*, 18 cells), hypoblast (hypo, 29 cells), yolk endoderm (yolk, 53 cells), ectoderm (ectoderm 29 cells), hemogenic endothelium (hemo-endo. 37 cells), myeloid progenitor (mye-prog. 17 cells), erythro-myeloid progenitor (ery-my p. 28 cells), blood progenitor (blood prog. 29 cells), erythrocyte (erythro. 32 cells). **d,** Cell-type-specific expression of TE subfamilies; heatmap of the -log10 adjusted p-value of cell-type-specificity of TE subfamily expression; only TE subfamilies with adjusted p-value $< 10^{e-5}$ were plotted (p-value are established using non-parametric Wilcoxon rank sum test).

Fig. S2: Evolutionarily recent cell type-specific TEs have cis-regulatory potential during human development

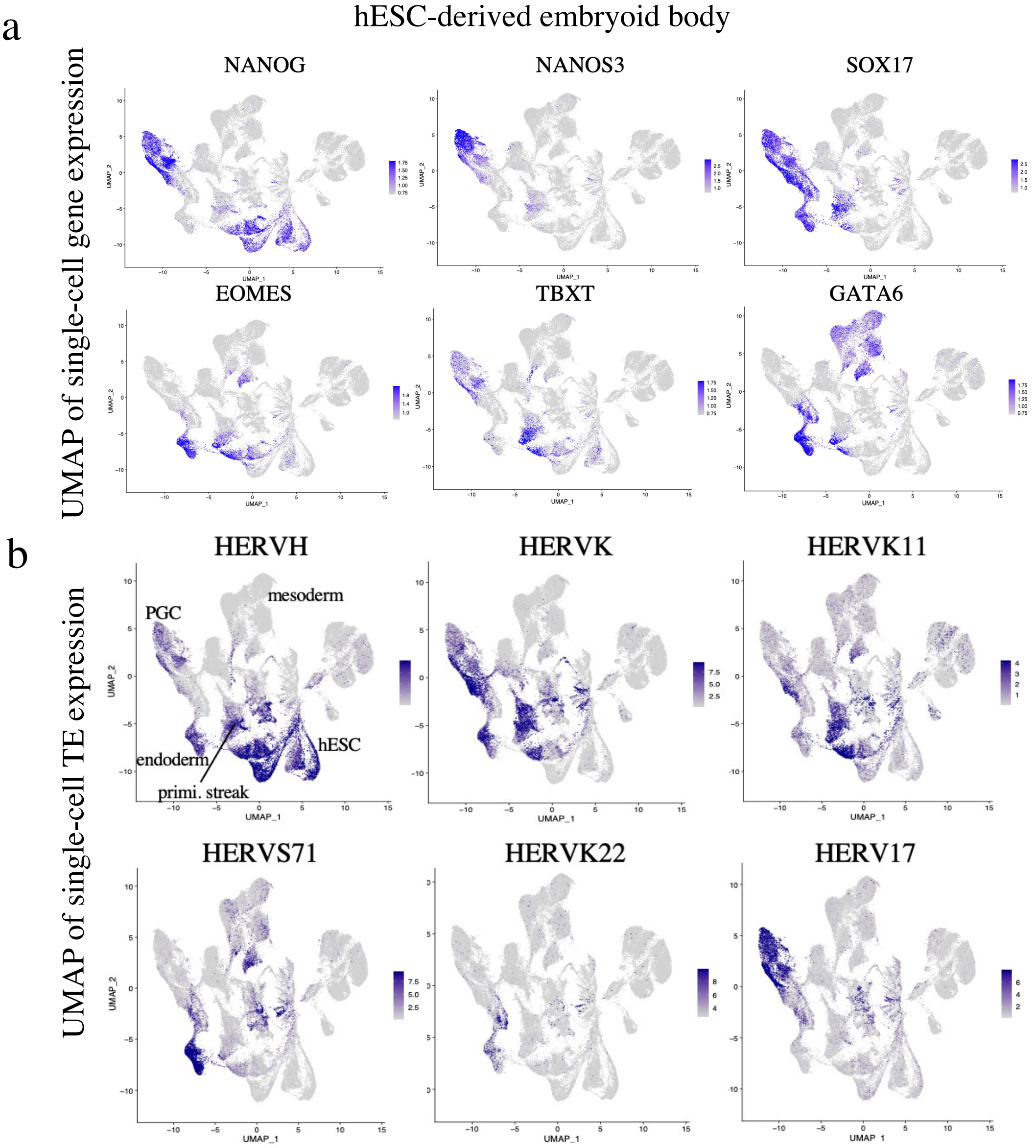

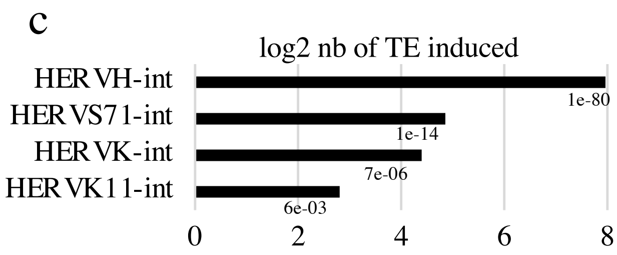

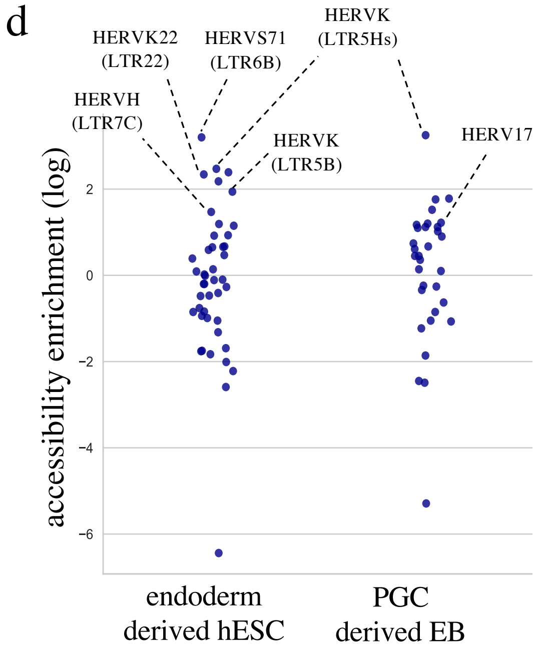

Fig. S2: Evolutionarily recent cell type-specific TEs have cis-regulatory potential during human development

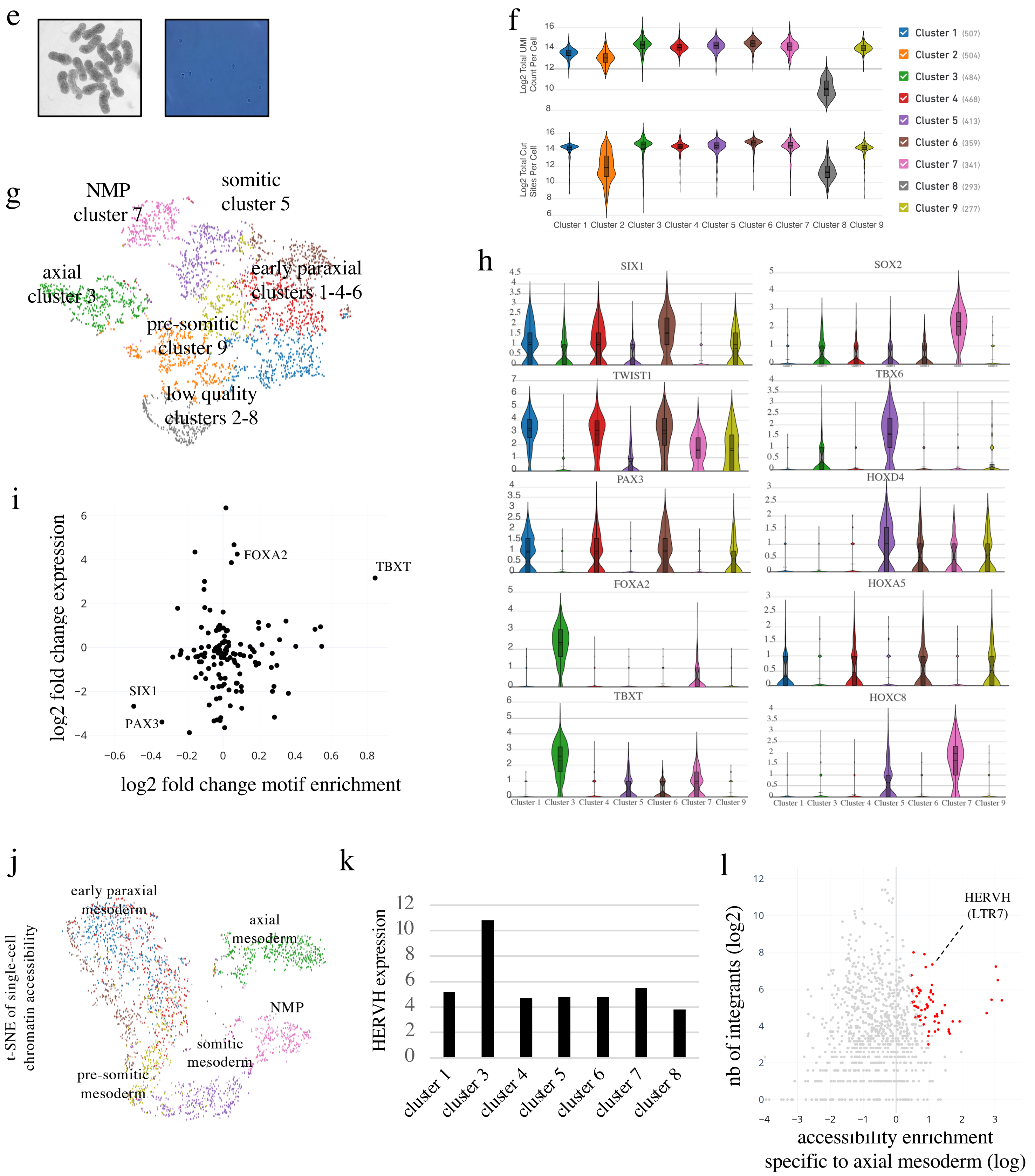

Figure S2 for Fig 2. TEs are controlled by tissue-specific transcription factors

a, Cell-type-specific expression of transcription factors during embryoid body differentiation. Each plot represents a UMAP defined on single-cell gene expression during differentiation of hESC into embryoid bodies over 5 days, re-analyzed from[26]; hESC corresponds to day 0; primitive streak (prim. streak) to days 1-2 with TBXT expression; cells at days 2-5 are stratified into PGC expressing NANOS3, NANOG, and SOX17, endoderm expressing SOX17 and GATA6, and mesoderm expressing GATA6 only. Color scale corresponds to level of relative TE subfamily expression based on normalized read counts. **b,** Cell-type-specific expression of TE subfamily during embryoid body differentiation. Each plot represents a UMAP of single-cell gene expression during *in vitro* differentiation of hESC into embryoid body over 5 days re-analyzed from[26]; hESC corresponds to day 0; primitive streak (prim. streak) to days 1-2 with TBXT expression; cells at days 2-5 are stratified in PGC expressing NANOS3, NANOG, and SOX17, endoderm expressing SOX17 and GATA6, and mesoderm expressing GATA6 only. Color scale corresponds to level of relative TE subfamily expression based on normalized read counts. **c,** Log2 number of expressed TE integrants induced upon hESC-derived endodermal differentiation; p-value enrichment is represented for each presented subfamily (two-sided t.test). **d,** Chromatin accessibility at endodermal and PGC-expressed TE subfamilies (adjusted p-value < 0.05) in hESC-derived endoderm and PGC, calculated over random genomic distribution and represented in natural log. **e,** Gastruloid formation and nuclei isolation. Left panel, picture of pooled elongated gastruloids used for the multi-omics experiment; right panel, trypan blue of purified nuclei used for the multi-omics experiment. **f,** UMI (unique molecular identifier) distribution of gene expression and transposase cut site counts of chromatin accessibility in each cluster defined by expression in single-nuclei RNA-seq and ATAC-seq. Clusters 2 and 8 contained lower amounts of RNA and/or ATAC-seq UMI/cut sites, hence were ignored for the rest of the analysis; clusters 1 and 4 look similar to cluster 6 but additionally express different cycling genes. Each cluster from 1-9 contains 507, 504, 484, 468, 413, 359, 341, 293, 277 cells respectively. **g,** UMAP of single-cell clustering of gastruloids based on gene expression; each color represents a different cluster illustrated in Fig. S2c. **h,** Violin plot of expression level of indicated cell-type-specific transcription factors for each cluster from Fig. S2c; cell-type-specific transcription factors were selected based on cluster-specificity of expression and DNA binding motif enrichment for their chromatin accessibility. Each cluster from 1-9 contains 507, 504, 484, 468, 413, 359, 341, 293, 277 cells respectively. **i,** Relative expression (*y-axis*) and motif enrichment at accessible chromatin (*x-axis*) of cell-type-specific transcription factors in axial mesodermal cells (cluster 3) comparted with other clusters.**j,** Chromatin accessibility in human gastruloids, analyzed at the single-cell level. t-SNE plot (t-distributed Stochastic Neighbor Embedding) representing chromatin accessibility clustering. Colors correspond to gene expression clustering from Fig. S2c-e. **k**, Expression profile of the HERVH subfamily in gastruloid-derived clusters. The *y-axis* corresponds to the normalized sum of accessible HERVH expression from axial mesoderm (315 loci) in each cluster. **l**, TE subfamily enrichment of chromatin accessibility (p-value < 0.05) in axial mesoderm. Each dot represents a TE subfamily*; x-axis*, natural log fold enrichment of TE loci compared to a random genomic distribution and *y-axis* represents the log2 number of accessible integrants. Red dots are TE subfamilies with a p-value of enrichment < 0.05.

Fig. S3: Tissue-specific transcription factors control cell-type-specificity of TE expression

a

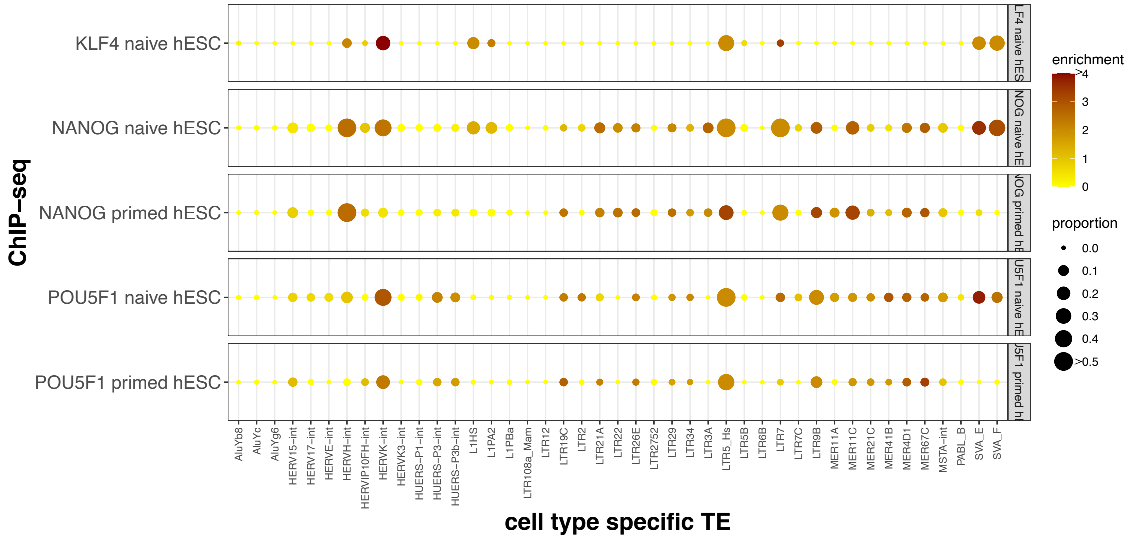

b

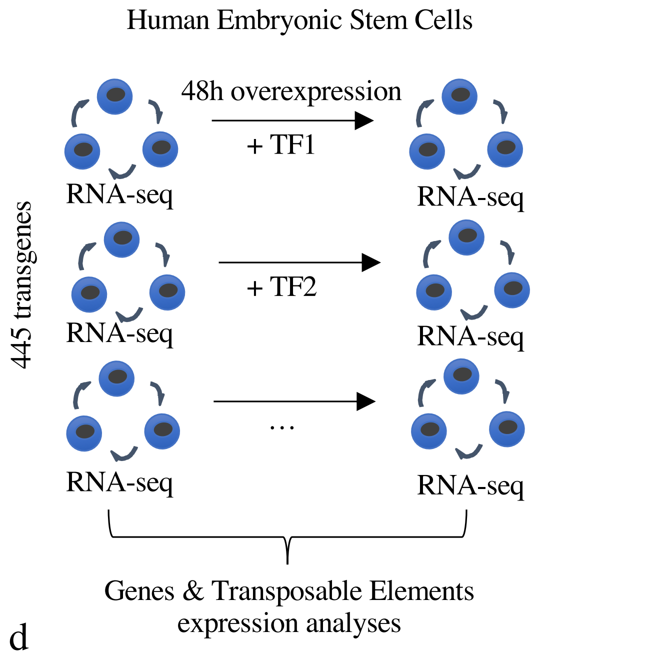

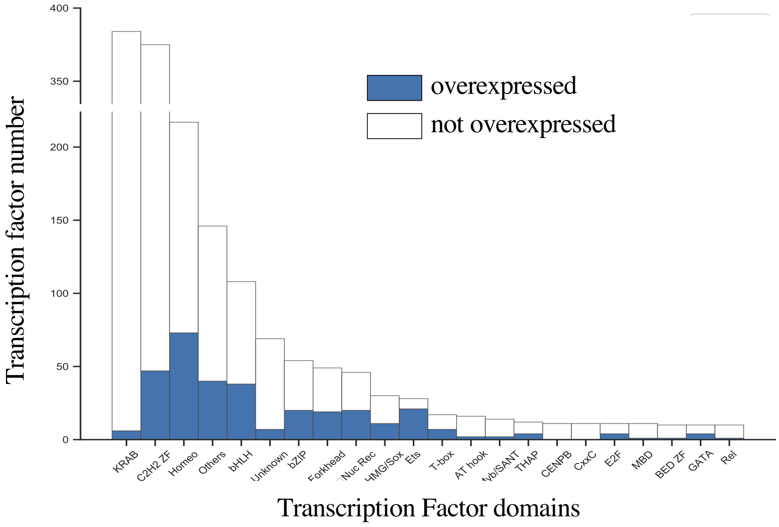

d

	coding genes	transposable elements
total	20'327	4'570'939
expressed	18'817	397'163
deregulated	18'428	216'160

e

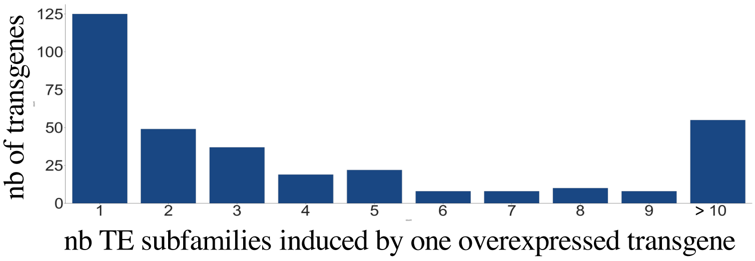

f

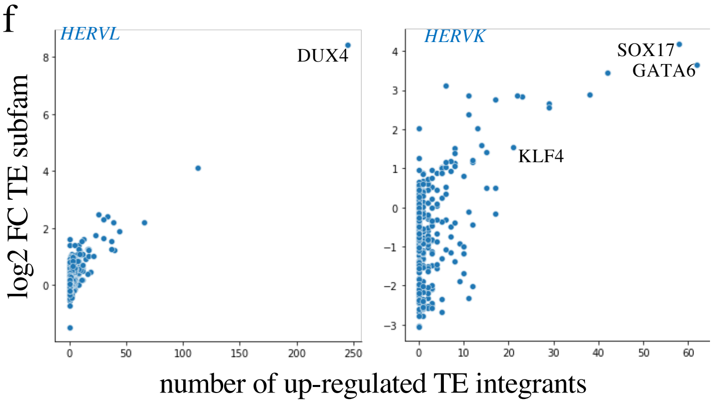

g

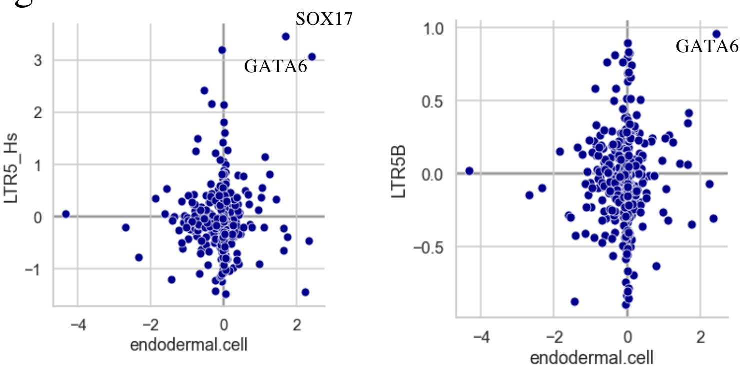

Figure S3 for Fig 3. Evolutionary recent TEs act as cell-type-specific enhancers during human gastrulation and fetal development.

a, Binding of pluripotency factors at TE expressed in gastrula and accessible in pre-implantation *in vitro* or *in vitro* models. The *x-axes* correspond to the cell type-specific TE subfamilies in human gastrula (p.value < 10e-5) that are also accessible in one of the in vivo or in vitro models (p.value < 10e-3), the *y-axis* the ChIP-seq of pluripotency factors in primed/naïve hESCs; circle sizes represent the number of accessibility sites overlapping with a specific TE subfamily, normalized by the number of elements in that subfamily (p-value are established using Homer algorithm); color intensity represents the log enrichment relative to the random distribution of this overlap. **b,** Design of TF overexpression in hESC experiment performed in[31]. **c,** Overexpressed TF subtypes. Each bar represents the number of proteins harboring the indicated domain, with fraction in blue indicating those overexpressed in [31]. **d,** Sum of total, expressed and differentially expressed genes and TEs upon overexpression of 234 TFs in hESC (with adjusted p-value <0.05, two-sided t.test with p.value correction for multiple testing using the Benjamini-Hochberg's method). **e**, Number of TE subfamilies deregulated per overexpressed TF; each bar plot represents the number of TFs inducing the number of TE subfamilies as indicated on *x-axis* (adjusted p-value < 0.05 for significantly up-regulated TEs over-representation in a TE subfamily, two-sided t.test with p.value correction for multiple testing using the Benjamini-Hochberg's method). **f,** Scatter plot illustrating coupling between EGA-induced TFs and TEs. *y-axis*, log2 fold TE subfamily add-up of normalized read count expression change; *x-axis*, number of up-regulated TE integrants from that subfamily (2-fold with adjusted p-value <0.05, two-sided t.test with p.value correction for multiple testing using the Benjamini-Hochberg's method). **g,** Scatter plot illustrating correlation between germ layer-specific TEs and TFs. *y-axis*, log2 fold TE subfamily add-up of normalized read count expression induced by overexpressed transcription factors in hESC; *x-axis*, log2 fold change expression of these transcription factors in human gastrula versus epiblast cells.

Fig. S4: Cell type-specific TE control gene expression during gastrulation

a

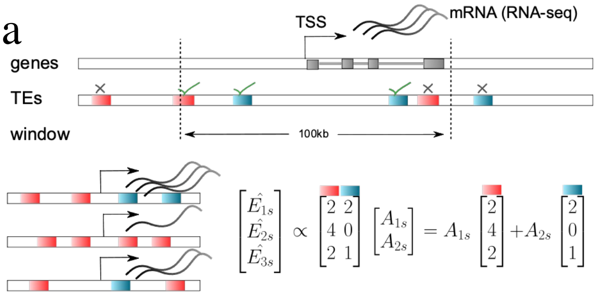

b

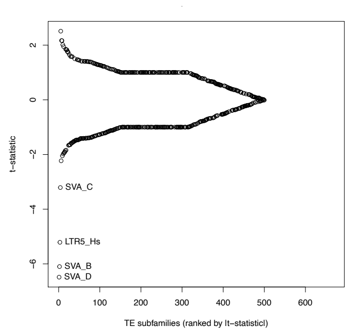

c

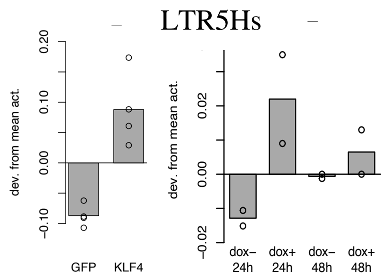

d

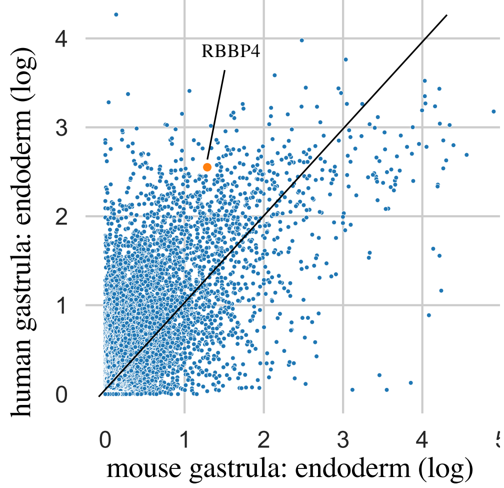

e

TE subfamilly name	number of integrants	predicted targets
LTR5_Hs	645	483
LTR5B	431	178
LTR5A	265	1
LTR5	20	2

f

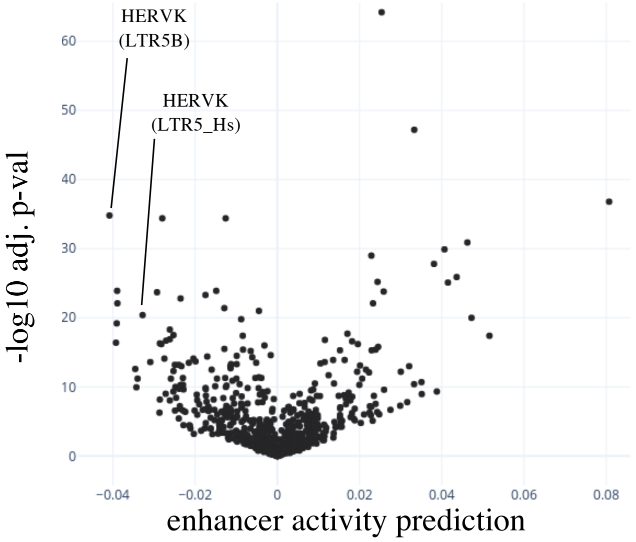

g

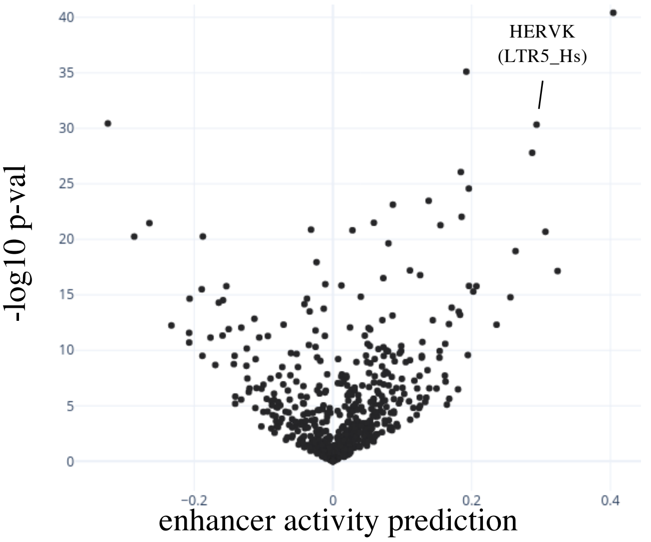

h

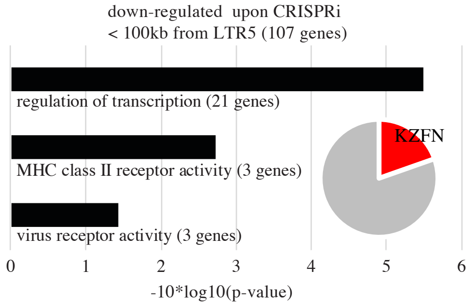

Figure S4 for Fig 4. Cell-type-specific TE control gene expression during gastrulation
a, TE subfamily-derived enhancer activity estimation schema. Regressive correlation is applied based on each coding gene expression level (Ex) and neighboring representation (100kb around TSS) of each TE subfamilies (T) in each TF-overexpressing hESC (E), allowing estimation of each TE subfamily relative enhancer activity (A). **b,** T-statistical tests of TE subfamily activity around deregulated genes upon CRISPRi-targeting SVA/LTR5Hs in naïve hESCs. **c,** Activity of LTR5Hs subfamily in the presence or absence of KLF4 overexpression in primed hESCs from[9], (left), and[31] at 24h or 48h overexpression (right). **d,** Scatterplot of gene expression comparison of human and mouse endoderm. Red dot highlights the *RBBP4* expression level; single-cell RNA-seq expression data of endodermal tissues of human gastrula[25] is compared to a single-cell RNA-seq of endodermal tissues of mouse gastrula (mixed stages) [70]. **e,** Predicted number of LTR5 loci targeted by CRISPRi (identified by CRISPOR[53]). **f,** TE-derived enhancer activity prediction upon CRISPRi-targeting LTR5 during endodermal differentiation. Representation of enhancer activity prediction for all TE subfamilies after comparing the transcriptome of hESC-derived endodermal cells with or without CRISPRi targeting LTR5 after 3 days of differentiation*; x-axis* represents the activity value, and *the y-axis* the -log10 adjusted p-value (establish by null significance hypothesis testing on the linear regression coefficients and accounted for multiple testing using the Benjamini Hochberg procedure). **g,** TE-derived enhancer activity prediction between human and mouse endodermal gastrulating cells. Representation of enhancer activity prediction for all TE subfamilies after comparing single-cell RNA-seq expression data of endodermal tissues of human gastrula[25] and the single-cell RNA-seq of endodermal tissues of mouse gastrula (mixed stages)[70]; *x-axis* represents the activity value and the *y-axis* the -log10 p-value (establish by null significance hypothesis testing on the linear regression coefficients). **h,** Gene Ontology of nearby LTR5-controlled genes. All down-regulated genes upon LTR5-targeting CRISPRi in a 100kbp window from LTR5 were selected; 2D-pie represents the proportion of 21 KZNF genes among the 107 down-regulated genes.

Fig. S5: Primate specific cis- and trans-regulators partner up to control human gastrulation

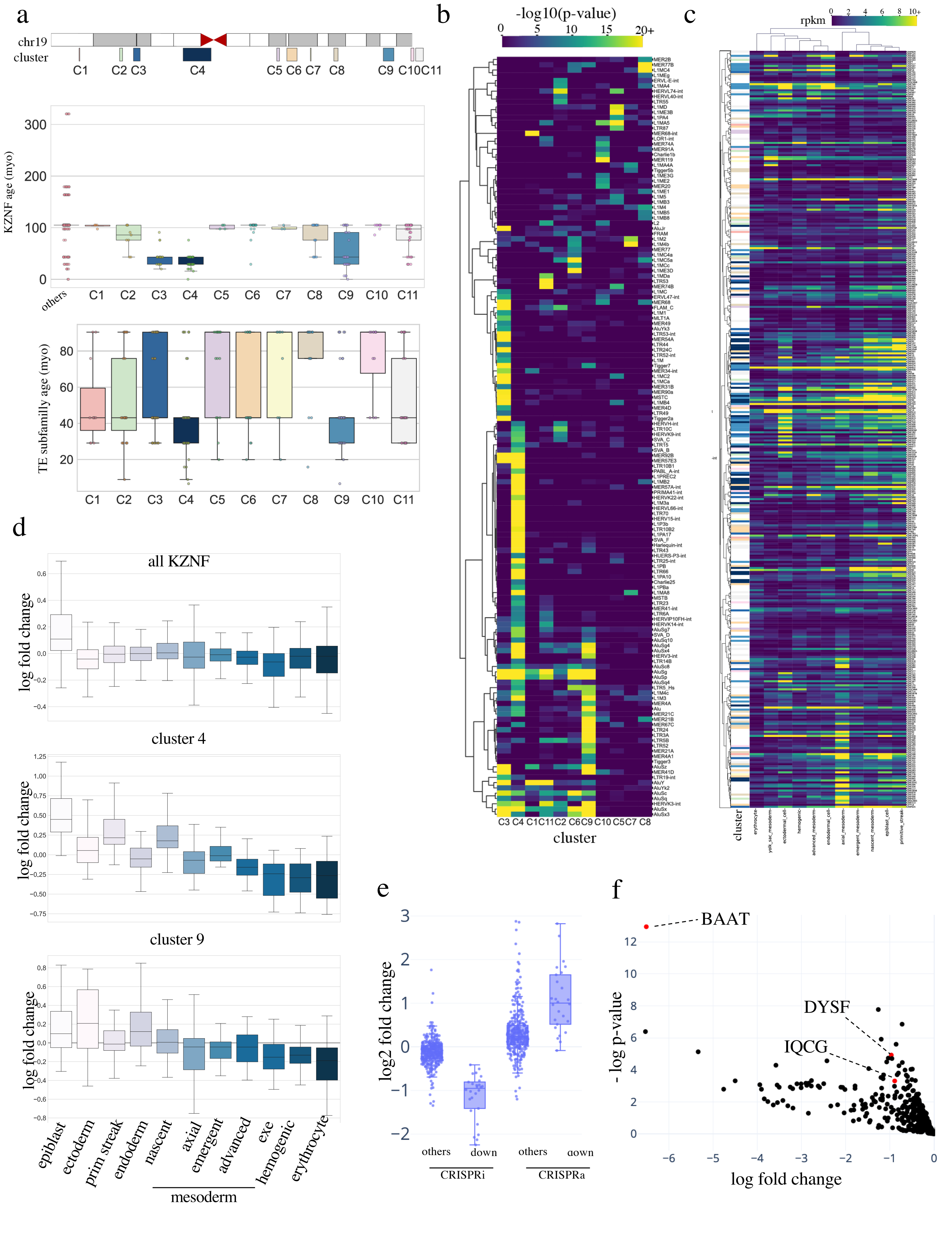

Figure S5 for Fig 5. Primate specific cis- and trans-regulators partner up to control human gastrulation

a, Evolutionary young *KZFP* gene clusters are enriched in contemporary TEs. Top, boxplot of *KZFP* genes evolutionary ages within 11 clusters (C1 to C11, with 4, 9, 20, 33, 5, 28, 3, 18, 37, 9, 39 genes respectively) on chromosome 19 or elsewhere in the genome (others with 152 genes); bottom, boxplot of evolutionary ages of TE subfamilies enriched in these *KZFP* gene clusters (p-value < 10^{e-4}, with 8, 19, 39, 58, 10, 17, 4, 10, 28, 8, 17 TE subfamilies respectively from C1 to C11).

b, Heatmap of TE subfamily enrichment in *KZFP* gene clusters. Yellow intensity is proportional to significance (-10*log10(p-value established using Homer algorithm); only TE subfamily with a p-value enrichment of 10e-4, a fold change of 2 and at least 5 integrant inside one cluster were represented. **c**, Heatmap of *KZFP* genes expression in human gastrula re-analyzed from[67]. Yellow intensity is proportional to log normalized counts; only *KZFP* genes with at least 2 normalized counts were plotted.

d, *KZFP* genes expression in human gastrula. Each boxplot represents log2 gene expression fold change of one cell type over the others (386 *KZFP* genes). Top panel shows the expression of all *KZFP* genes; middle and bottom panels depict *KZFP* genes in clusters 4 and 9, respectively. **e,** LTR5-controlled *KZFP* genes vs other genes during endodermal differentiation are activated upon CRISPRa in NCCIT cell line. Left, boxplots of *KZFP* genes significantly (down, 26 genes) or not (others, 339 genes) down-regulated upon LTR5-mediated repression during endodermal differentiation; on the right are presented the fold change upon LTR5-mediated activation in NCCIT cell line of the down (26 genes) and others (339 genes) KZFPs re-analyzed from[72]. **f,** Proximal MER11 genes downregulated upon CRISPRi targeting of LTR5 in hESC-derived foregut. The *x-axis* represents the logarithmic change; the *y-axis* represents the logarithmic p-value (two-sided t.test). Only genes located within 50kb of a MER11 and downregulated are shown. Red dots represent genes identified in in[35].

Observed/Expected extracted from Barakat et al. 2018	Primed hESC					Naive hESC				
TE subfamillies	p_min-128	p_128-256	p_256-512	p_512-1024	p_1024-max	n_min-128	n_128-256	n_256-512	n_512-1024	n_1024-max
LTR7	0.79	1.46	2.76	4.99	8.90	0.95	0.80	0.00	13.66	0.00
LTR21A	0.99	1.25	1.58	0.00	0.00	0.80	2.27	2.10	8.57	0.00
MER11C	0.92	1.10	2.12	3.72	2.45	0.89	1.35	3.76	0.00	0.00
MER4D1	0.95	1.29	1.16	2.20	2.11	0.86	1.92	2.50	2.56	0.00
MER67C	0.93	0.72	1.49	3.80	4.31	0.96	1.40	1.55	0.00	0.00
L1PA2	0.92	1.52	2.33	1.47	1.91	0.96	1.31	1.31	1.55	0.69
LTR34	0.91	2.47	0.70	0.00	2.78	0.96	0.00	5.41	0.00	0.00
MER11A	0.97	0.81	1.23	4.27	0.82	0.93	1.69	1.39	1.06	0.00
MSTA-int	0.91	1.06	2.11	3.00	3.84	1.04	1.03	0.00	0.00	0.00
LTR29	0.96	0.91	1.53	0.80	3.83	1.00	0.79	1.47	1.50	0.00
LTR5_Hs	0.95	0.72	1.55	3.13	3.59	1.04	0.74	0.62	0.00	0.00
LTR9B	0.94	1.15	1.81	0.87	3.62	1.05	0.51	0.86	1.17	0.00
LTR26E	0.92	1.03	4.59	0.00	1.31	0.91	2.03	1.13	0.00	0.00
LTR6B	0.96	0.79	1.00	0.00	5.99	1.02	0.83	1.15	0.00	0.00
LTR3A	0.99	0.00	1.70	0.00	6.81	1.03	1.15	0.00	0.00	0.00
HERVH-int	0.95	1.01	1.27	2.17	3.34	1.04	0.48	1.32	0.00	0.00
LTR5B	1.01	0.81	1.03	1.07	1.03	0.98	1.12	0.78	3.17	0.00
HUERS-P3b-int	1.03	0.94	0.79	0.00	0.00	0.95	0.83	2.32	3.79	0.00
AluYg6	1.04	0.70	0.89	0.00	0.00	0.87	2.25	0.89	3.64	0.00
LTR12	1.00	0.58	1.48	3.09	0.00	1.01	0.44	2.46	0.00	0.00
HERVIP10FH-int	1.04	0.81	0.68	0.00	0.45	1.02	0.36	1.51	4.11	0.00
LTR7C	1.04	0.39	1.00	2.08	0.00	1.00	1.23	0.44	1.04	1.50
LTR2	1.01	0.84	0.35	0.74	2.83	0.97	1.33	1.23	0.00	0.00
MER41B	0.98	1.10	0.85	2.11	1.69	1.07	0.68	0.00	0.00	0.00
SVA_E	1.05	0.43	0.79	0.61	0.20	0.93	1.66	1.15	1.57	0.00
L1HS	0.97	1.24	1.33	0.81	1.44	1.07	0.34	0.94	0.00	0.00
HUERS-P1-int	1.07	0.00	0.81	0.00	1.61	0.91	1.88	1.74	0.00	0.00
HERV17-int	1.02	1.05	0.45	0.93	0.00	0.86	2.79	0.78	0.00	0.00
MER21C	1.00	0.77	1.13	1.23	1.42	1.06	0.62	0.58	0.00	0.00
LTR19C	0.97	1.36	1.15	2.39	0.00	1.07	0.56	0.19	0.00	0.00
L1PBa	1.01	0.63	1.34	1.02	0.98	1.07	0.27	1.14	0.00	0.00
LTR22	0.98	1.06	2.70	0.00	0.00	0.98	1.68	0.00	0.00	0.00
PABL_B	1.04	0.00	1.39	2.90	0.00	1.09	0.31	0.28	0.00	0.00
HERVK-int	1.01	0.65	0.99	2.75	0.00	1.11	0.13	0.36	0.00	0.00
AluYc	1.00	1.03	0.98	0.87	0.84	1.07	0.62	0.00	0.00	0.00
HERVE-int	1.02	0.72	1.23	0.00	1.23	1.06	0.80	0.00	0.00	0.00
HERVK3-int	1.07	0.67	0.00	0.00	0.00	0.95	1.43	1.70	0.00	0.00
HUERS-P3-int	1.00	1.47	0.62	0.00	0.00	1.09	0.49	0.00	0.00	0.00
SVA_F	1.08	0.25	0.36	0.56	0.24	1.06	0.61	0.31	0.00	0.00
AluYb8	1.06	0.47	0.30	0.62	0.30	1.09	0.41	0.00	0.00	0.00
HERV15-int	1.11	0.00	0.00	0.00	0.00	1.00	1.03	1.07	0.00	0.00

Supplementary Table1. Enhancer activity of TE subfamily in Human Embryonic Stem cells

Data extracted from Barakat et al. 2018 supplemental data in primed and naive human embryonic stem cells (hESC). The most accessible subfamilies in Figure 2A were selected and their enhancer activity represented in this table when tested in hESCs with an episomal reporter assay.
