# Supplementary material for: Primate-specific transposable elements shape transcriptional networks during human development
Source: Nat Commun. 2022 Nov 23;13:7178. doi: 10.1038/s41467-022-34800-w (PMC9684439; doi:10.1038/s41467-022-34800-w)
Supplement: Supplementary file 2 — Description of Additional Supplementary Files [file 41467_2022_34800_MOESM2_ESM.pdf]

Description of Additional Supplementary Files

File Name: Supplementary Data 1
Description: Matching the binding profiles of 268 TFs as defined in various cellular contexts[32] with transcriptome studies performed in overexpressing hESCs[31]. 156 factors that could bind to and induce the expression of 667 TE subfamilies are represented in the table.
