# Supplementary material for: Primate-specific transposable elements shape transcriptional networks during human development
Source: Nat Commun. 2022 Nov 23;13:7178. doi: 10.1038/s41467-022-34800-w (PMC9684439; doi:10.1038/s41467-022-34800-w)
Supplement: Supplementary file 4 — Reporting Summary [file 41467_2022_34800_MOESM4_ESM.pdf]

nature portfolio

Corresponding author(s): Julien Pontis and Didier Trono

Last updated by author(s): Jul 9, 2022

Reporting Summary

Nature Portfolio wishes to improve the reproducibility of the work that we publish. This form provides structure for consistency and transparency in reporting. For further information on Nature Portfolio policies, see our Editorial Policies and the Editorial Policy Checklist.

Statistics

For all statistical analyses, confirm that the following items are present in the figure legend, table legend, main text, or Methods section.

n/a	Confirmed	
☐	☒	The exact sample size (*n*) for each experimental group/condition, given as a discrete number and unit of measurement
☐	☒	A statement on whether measurements were taken from distinct samples or whether the same sample was measured repeatedly
☐	☒	The statistical test(s) used AND whether they are one- or two-sided<br>*Only common tests should be described solely by name; describe more complex techniques in the Methods section.*
☒	☐	A description of all covariates tested
☐	☒	A description of any assumptions or corrections, such as tests of normality and adjustment for multiple comparisons
☐	☒	A full description of the statistical parameters including central tendency (e.g. means) or other basic estimates (e.g. regression coefficient) AND variation (e.g. standard deviation) or associated estimates of uncertainty (e.g. confidence intervals)
	☒	For null hypothesis testing, the test statistic (e.g. *F*, *t*, *r*) with confidence intervals, effect sizes, degrees of freedom and *P* value noted<br>*Give P values as exact values whenever suitable.*
☒	☐	For Bayesian analysis, information on the choice of priors and Markov chain Monte Carlo settings
☒	☐	For hierarchical and complex designs, identification of the appropriate level for tests and full reporting of outcomes
☒	☐	Estimates of effect sizes (e.g. Cohen's *d*, Pearson's *r*), indicating how they were calculated

Our web collection on statistics for biologists contains articles on many of the points above.

Software and code

Policy information about availability of computer code

Data collection	FlowJo LLC - v8.8.7, IGV - v2.8.4, Homer - v4.10.4, UCSC liftOver tool, Bowtie2 - v2.27.1, Samtools - v1.10, MACS2 - 2.2.4, Bedtools - v2.27.1, HISAT2 - v2.1.0, R - v4.1.2, Bioconductor 3.13, Seurat v4.1.0, ggplots v3.1.1 , GenomicRanges - v1.48.0, pyBigWig - v0.3.18, CRISPOR - v5.01, deeptools - v3.5.1, featureCounts v1.6.2
Data analysis	https://github.com/julienpontis/TE-Gastrulation

For manuscripts utilizing custom algorithms or software that are central to the research but not yet described in published literature, software must be made available to editors and reviewers. We strongly encourage code deposition in a community repository (e.g. GitHub). See the Nature Portfolio guidelines for submitting code & software for further information.

Data

Policy information about availability of data

All manuscripts must include a data availability statement. This statement should provide the following information, where applicable:

- Accession codes, unique identifiers, or web links for publicly available datasets
- A description of any restrictions on data availability
- For clinical datasets or third party data, please ensure that the statement adheres to our policy

- GEO for this study: GSE181120
- Re-analysed datasets GSE140021, GSE120648, GSE117136, GSE52657, GSE130418, E-MTAB-9388, DRA006296
- No restriction for data availability

Human research participants

Policy information about studies involving human research participants and Sex and Gender in Research.

Reporting on sex and gender	*Use the terms sex (biological attribute) and gender (shaped by social and cultural circumstances) carefully in order to avoid confusing both terms. Indicate if findings apply to only one sex or gender; describe whether sex and gender were considered in study design whether sex and/or gender was determined based on self-reporting or assigned and methods used. Provide in the source data disaggregated sex and gender data where this information has been collected, and consent has been obtained for sharing of individual-level data; provide overall numbers in this Reporting Summary. Please state if this information has not been collected. Report sex- and gender-based analyses where performed, justify reasons for lack of sex- and gender-based analysis.*
Population characteristics	*Describe the covariate-relevant population characteristics of the human research participants (e.g. age, genotypic information, past and current diagnosis and treatment categories). If you filled out the behavioural & social sciences study design questions and have nothing to add here, write "See above."*
Recruitment	*Describe how participants were recruited. Outline any potential self-selection bias or other biases that may be present and how these are likely to impact results.*
Ethics oversight	*Identify the organization(s) that approved the study protocol.*

Note that full information on the approval of the study protocol must also be provided in the manuscript.

Field-specific reporting

Please select the one below that is the best fit for your research. If you are not sure, read the appropriate sections before making your selection.

☒ Life sciences ☐ Behavioural & social sciences ☐ Ecological, evolutionary & environmental sciences

For a reference copy of the document with all sections, see nature.com/documents/nr-reporting-summary-flat.pdf

Life sciences study design

All studies must disclose on these points even when the disclosure is negative.

Sample size	All experiments contains at least 2 replicates (for RNA-seq) and more than 3 for ChIP and RT-qPCR per data point were performed.
Data exclusions	No data were excluded
Replication	All experiments contains at least 2 replicates (for RNA-seq) and more than 3 for ChIP and RT-qPCR per data point were performed. All replicates were integrated in all figures.
Randomization	No randomization were applicable
Blinding	3 different persons performed all the experiments at different steps, and in each cases the person performing RNA-seq library prep, its raw analysis as well as qPCR didn't know which sample were the controls.

Reporting for specific materials, systems and methods

We require information from authors about some types of materials, experimental systems and methods used in many studies. Here, indicate whether each material, system or method listed is relevant to your study. If you are not sure if a list item applies to your research, read the appropriate section before selecting a response.

Materials & experimental systems

n/a	Involved in the study	
☒	☐	Antibodies
☐	☒	Eukaryotic cell lines
☒	☐	Palaeontology and archaeology
☒	☐	Animals and other organisms
☒	☐	Clinical data
☒	☐	Dual use research of concern

Methods

n/a	Involved in the study	
☒	☐	ChIP-seq
☐	☒	Flow cytometry
☒	☐	MRI-based neuroimaging

Eukaryotic cell lines

Policy information about cell lines and Sex and Gender in Research

Cell line source(s)	The commonly used H1 (WAe001-A) and H9 (WAe009-A) human embryonic stem cell lines were originally derived from embryos produced using in vitro fertilization for reproduction and no longer needed for this purpose, and donated through voluntary written consent for use in research.
Authentication	Human Embryonic stem cells have a recognizable phenotype and their transcriptome correspond their corresponding transcriptionnal profile.
Mycoplasma contamination	cells were tested for mycoplasma and were negative
Commonly misidentified lines (See ICLAC register)	None of the cell line belong to commonly misidentified cell lines

Flow Cytometry

Plots

Confirm that:

- [x] The axis labels state the marker and fluorochrome used (e.g. CD4-FITC).
- [x] The axis scales are clearly visible. Include numbers along axes only for bottom left plot of group (a 'group' is an analysis of identical markers).
- [ ] All plots are contour plots with outliers or pseudocolor plots.
- [ ] A numerical value for number of cells or percentage (with statistics) is provided.

Methodology

Sample preparation	cells were wash and harvest using TRYPLE and directly processed by FACS
Instrument	Becton Dickinson FACScan
Software	Flowjo
Cell population abundance	5000 cells per sample
Gating strategy	no gating were performed on GFP value, and dentical gating applied on all sample using FSC-H and SSC-H.

- [ ] Tick this box to confirm that a figure exemplifying the gating strategy is provided in the Supplementary Information.
